# A missense mutation in *SNRPE* linked to non-syndromal microcephaly interferes with U snRNP assembly and pre-mRNA splicing

**DOI:** 10.1371/journal.pgen.1008460

**Published:** 2019-10-31

**Authors:** Tao Chen, Bin Zhang, Thomas Ziegenhals, Archana B. Prusty, Sebastian Fröhler, Clemens Grimm, Yuhui Hu, Bernhard Schaefke, Liang Fang, Min Zhang, Nadine Kraemer, Angela M. Kaindl, Utz Fischer, Wei Chen

**Affiliations:** 1 Laboratory for Functional Genomics and Systems Biology, Berlin Institute for Medical System Biology, Max-Delbrück-Center for Molecular Medicine, Berlin, Germany; 2 Department of Biology, Southern University of Science and Technology (SUSTech), Shenzhen, China; 3 Cancer Science Institute of Singapore, National University of Singapore, Singapore; 4 Department of Biochemistry, Theodor-Boveri-Institute, University of Würzburg, Würzburg, Germany; 5 Academy for Advanced Interdisciplinary Studies, Southern University of Science and Technology (SUSTech), Shenzhen, China; 6 Charité-Universitätsmedizin Berlin, Institute of Cell Biology and Neurobiology, Berlin, Germany; 7 Charité-Universitätsmedizin Berlin, Department of Pediatric Neurology, Berlin, Germany; 8 Charité-Universitätsmedizin Berlin, Center for Chronically Sick Children, Berlin, Germany; University of North Carolina at Chapel Hill, UNITED STATES

## Abstract

Malfunction of pre-mRNA processing factors are linked to several human diseases including cancer and neurodegeneration. Here we report the identification of a *de novo* heterozygous missense mutation in the *SNRPE* gene (c.65T>C (p.Phe22Ser)) in a patient with non-syndromal primary (congenital) microcephaly and intellectual disability. *SNRPE* encodes SmE, a basal component of pre-mRNA processing U snRNPs. We show that the microcephaly-linked SmE variant is unable to interact with the SMN complex and as a consequence fails to assemble into U snRNPs. This results in widespread mRNA splicing alterations in fibroblast cells derived from this patient. Similar alterations were observed in HEK293 cells upon SmE depletion that could be rescued by the expression of wild type but not mutant SmE. Importantly, the depletion of SmE in zebrafish causes aberrant mRNA splicing alterations and reduced brain size, reminiscent of the patient microcephaly phenotype. We identify the *EMX2* mRNA, which encodes a protein required for proper brain development, as a major mis-spliced down stream target. Together, our study links defects in the *SNRPE* gene to microcephaly and suggests that alterations of cellular splicing of specific mRNAs such as *EMX2* results in the neurological phenotype of the disease.

## Author summary

In higher eukaryotes, the protein coding genes are first transcribed as precursor mRNAs (pre-mRNAs) and further processed by the spliceosome to form the mature mRNA for translation. Malfunction of pre-mRNA processing factors are linked to several human diseases including cancer and neurodegeneration. Here we report the identification of a *de novo* heterozygous missense mutation in the *SNRPE/SmE* gene in a patient with non-syndromal primary (congenital) microcephaly and intellectual disability. The effect of identified *de novo* mutation on SNRPE/SmE was characterized *in vitro*. The zebrafish was used as *in vivo* model to further dissect the physiological consequence and pathomechanism. Finally, the *EMX2* gene was identified as one of the major down stream target genes responsible for the phenotype. Our study links defects in the *SNRPE/SmE* gene to microcephaly and provides the new pathogenic mechanism for microcephaly.

## Introduction

In higher eukaryotes, the vast majority of protein-coding genes are transcribed as precursors (pre-mRNA) containing non-coding intronic and coding exonic sequences. These pre-mRNAs need to be extensively processed by splicing to generate the mature mRNA with an open reading frame. Splicing is mediated by macromolecular machines termed spliceosomes, which consist of five different small nuclear ribonucleoprotein (snRNP) subunits and a large number of additional protein cofactors [1–4]. The major spliceosome, containing U1, U2, U4, U5 and U6 snRNPs, is responsible for splicing of almost 99% of human pre-mRNAs whereas the minor spliceosome is required to excise a special class of very rare (ATAC) introns from certain mRNAs [5]. To generate mRNA variants with different coding potential, the splice sites (SSs) within pre-mRNAs are differentially utilized through alternative splicing (AS). This process occurs in >95% of human multi-exon genes, thus leading to a large increase of protein diversity [6–9]. The decision of AS is regulated through the cooperative interplay between *cis*-elements, including constitutive splicing elements (such as 5’ SSs, branch point (BP), polypyrimidine tract (PPT) and 3’ SSs) and optional *cis*-regulatory elements (exonic and intronic splicing enhancer/silencer called ESE, ESS, ISE, ISS), and *trans*-acting factors, such as core splicing machinery and splicing regulators (SR proteins and heterogenous ribonucleoproteins (hnRNPs)) [9–11]. It has been shown that AS plays critical roles in the specification of cell fates [12], tissue types [6,9], developmental process [13], sex determination [14] and stimulation response [15].

Due to the important role in regulation of gene expression and protein diversity, mRNA splicing is particularly sensitive to mutations and its dysregulation could lead to human diseases [16,17]. The most common type of mutations leading to aberrant splicing, are *cis*-acting mutations located in either constitutive splicing elements (5’ SS, 3’ SS and BP) or *cis*-regulatory elements (ESE, ESS, ISE and ISS) modulating spliceosome assembly [16]. For instance, ESE, ESS and 5’ SS mutations in the exon 10 of the MAPT gene, encoding the microtubule-associated protein Tau, have been identified as the cause of frontotemporal dementia with parkinsonism linked to chromosome 17 (FTDP-17) [18].

In addition to mutations affecting *cis*-elements, mutations in *trans*-acting splicing factors are also implicated in a set of human diseases. Since defects in these factors typically affect the splicing machinery as a whole, they affect the processing of many transcripts and hence often cause more complex etiologies than mutations in *cis* elements. An interesting example of this class are mutations in several protein components of U4/U6.U5 tri-small nuclear ribonucleoprotein (tri-snRNP) such as pre-mRNA processing factor 3 (PRPF3) [19], PRPF4 [20], PRPF6 [21], PRPF8 [22], PRPF31 [23,24] and SNRNP200 (also called BRR2) [25], that cause the autosomal dominant eye disease retinitis pigmentosa (adRP) [26]. In addition, mutations preventing the production of functional SMN protein cause spinal muscular atrophy (SMA) [27]. This protein is part of the SMN complex, which mediates the assembly of spliceosomal U snRNPs and hence determines the abundance of active spliceosomes. Although the SMN protein is ubiquitously expressed, the effect of SMN deficiency on the repertoire of snRNAs and aberrant splicing shows tissue specific dependence in a SMA mouse model [28]. In addition, mutations within SmB/B’ and SmE have been reported to be linked to cerebro-costo-mandibular syndrome (CCMS) [29,30] and hypotrichosis simplex (HS) [31], respectively. Although these mutations are identified as the genetic cause of these diseases, the disease etiologies are still unknown. Importantly, mutations in RNU4ATAC have been shown to affect the formation of minor spliceosome and cause Taybi-Linder syndrome/microcephalic osteodysplastic primordial dwarfism type 1 (TALS/MOPD1) [32,33], illustrating that not only malfunctioning of proteins but also of U snRNAs can cause disease.

Using whole exome sequencing, we report here a *de novo* heterozygous missense mutation within the *SNRPE/SmE* gene from a patient with non-syndromal primary (congenital) microcephaly and intellectual disability. This mutation generates a protein product that fails to interact with the SMN-complex and thus cannot become properly assembled into spliceosomal U snRNPs. Our results further reveal that the mutation in SmE causes aberrant mRNA splicing in both human cell lines (fibroblast and HEK293) and zebrafish. Furthermore, specific depletion of endogenous SmE protein in zebrafish causes similar brain defect as in the patient. Of note, we find that one of the affected transcripts in the zebrafish model encodes for the protein EMX2, which is required for proper early brain development. Our study suggest that the identified missense mutation in *SNRPE* disturbs appropriate spatiotemporal gene expression in the brain through aberrant mRNA splicing, which is likely to cause the microcephaly phenotype.

## Results

### Identification of a missense mutation within *SNRPE/SmE* in a microcephaly patient

**Fig 1:**
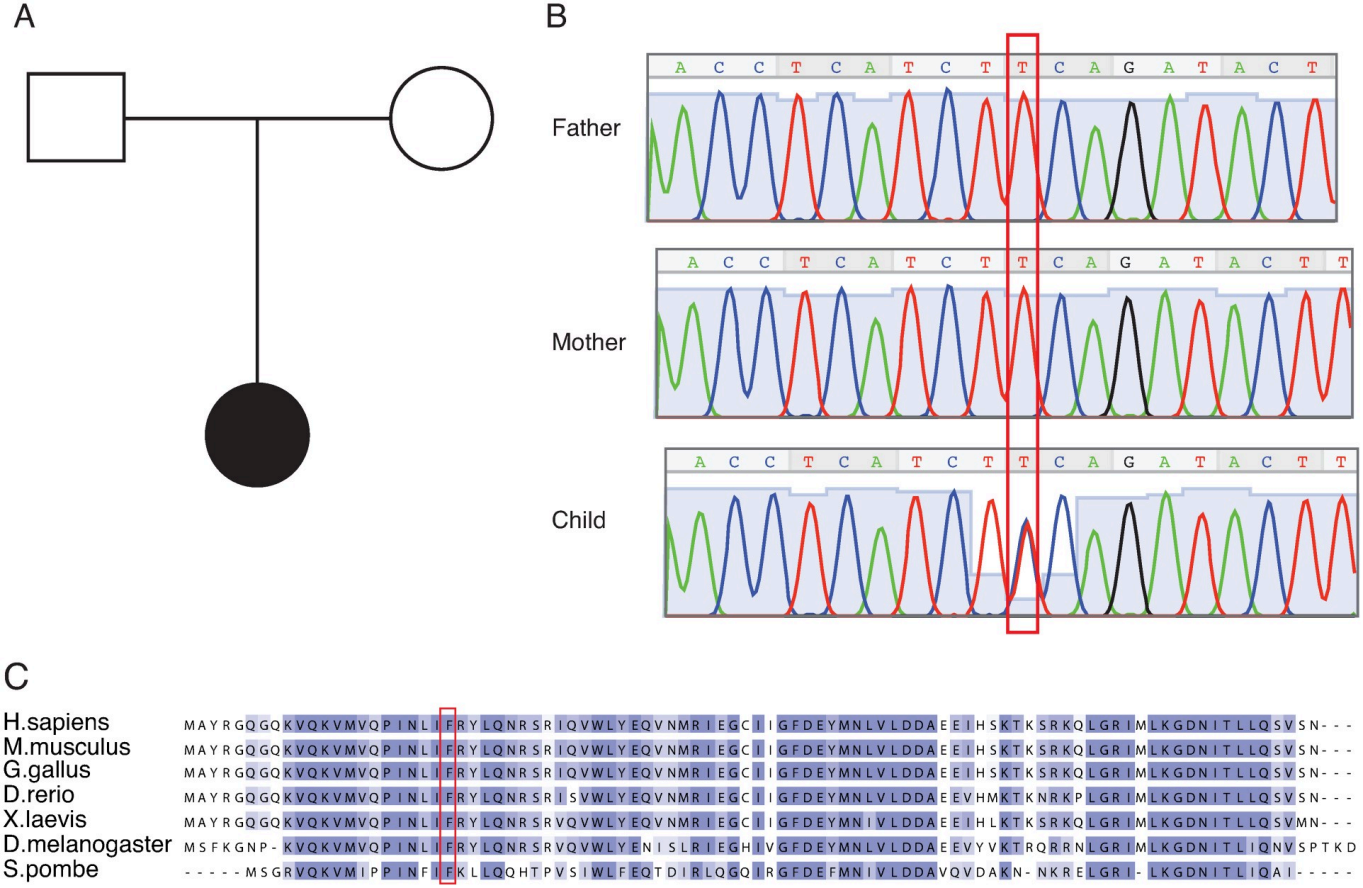
Identification of potential causative mutation by whole exome sequencing. (A), Family pedigree. Filled symbol indicates individual suffering from non-syndromal primary microcephaly and intellectual disability. (B), Traditional Sanger sequencing validated the identified *SNRPE/SmE* mutation (c.65T>C (p.Phe22Ser)). The red box labels the *de novo* heterozygous mutation. (C), Alignment of SNRPE/SmE protein sequences across different species. The red rectangle indicates the mutated residue.

To identify the molecular genetic basis of a patient afflicted with non-syndromal microcephaly in a two-generation pedigree, whole exome sequencing (WES) was performed for the patient and its unaffected parents (Fig 1A). On average, 180 million reads were obtained for each individual and more than 90 fold coverage of exome were achieved for each individual. A *de novo* heterozygous missense mutation (c.65T>C (p.Phe22Ser)) was identified in the second exon of the *SNRPE*/*SmE* gene from the patient (Fig 1B). This gene and in particular the mutated residue is highly conserved among different species including zebrafish and the more distant yeast *S*. *pombe* (Fig 1C). It encodes the SNRPE/SmE protein [34], which constitutes a basal component of spliceosome. This factor, together with six additional Sm proteins termed SmB/B’, SmD1, SmD2, SmD3, SmF and SmG, form the common Sm core of spliceosomal U snRNPs. This raised the possibility that the pathological mutation in SmE affects U snRNP biogenesis and/or splicing.

### Impaired binding of SmE mutant to the SMN complex causes defects in Sm core assembly

We first investigated whether the identified missense mutation in SmE affects its incorporation into U snRNPs. Incorporation of newly translated SmE starts with the formation of the heterotrimeric complex composed of SmE, SmF and SmG [35]. Subsequently, this heterooligomer is transferred onto the PRMT5 complex, which assembles together with SmD1/D2 and the assembly chaperone pICln a closed ring termed the 6S complex [36,37]. The next step of U snRNP biogenesis is dependent on the SMN complex, consisting of SMN, Gemins 2–8 and UNRIP [38]. This unit catalyzes the release of pICln from the 6S complex and the transfer of Sm proteins onto the U snRNA [36,37]. After hypermethylation of the m^7^G cap to m^2,2,7^_3_G (m_3_G/TMG) cap, the assembled U snRNPs are imported into the nucleus and after further maturation in Cajal bodies (CBs), targeted to splicing speckles [39,40].

**Fig 2:**
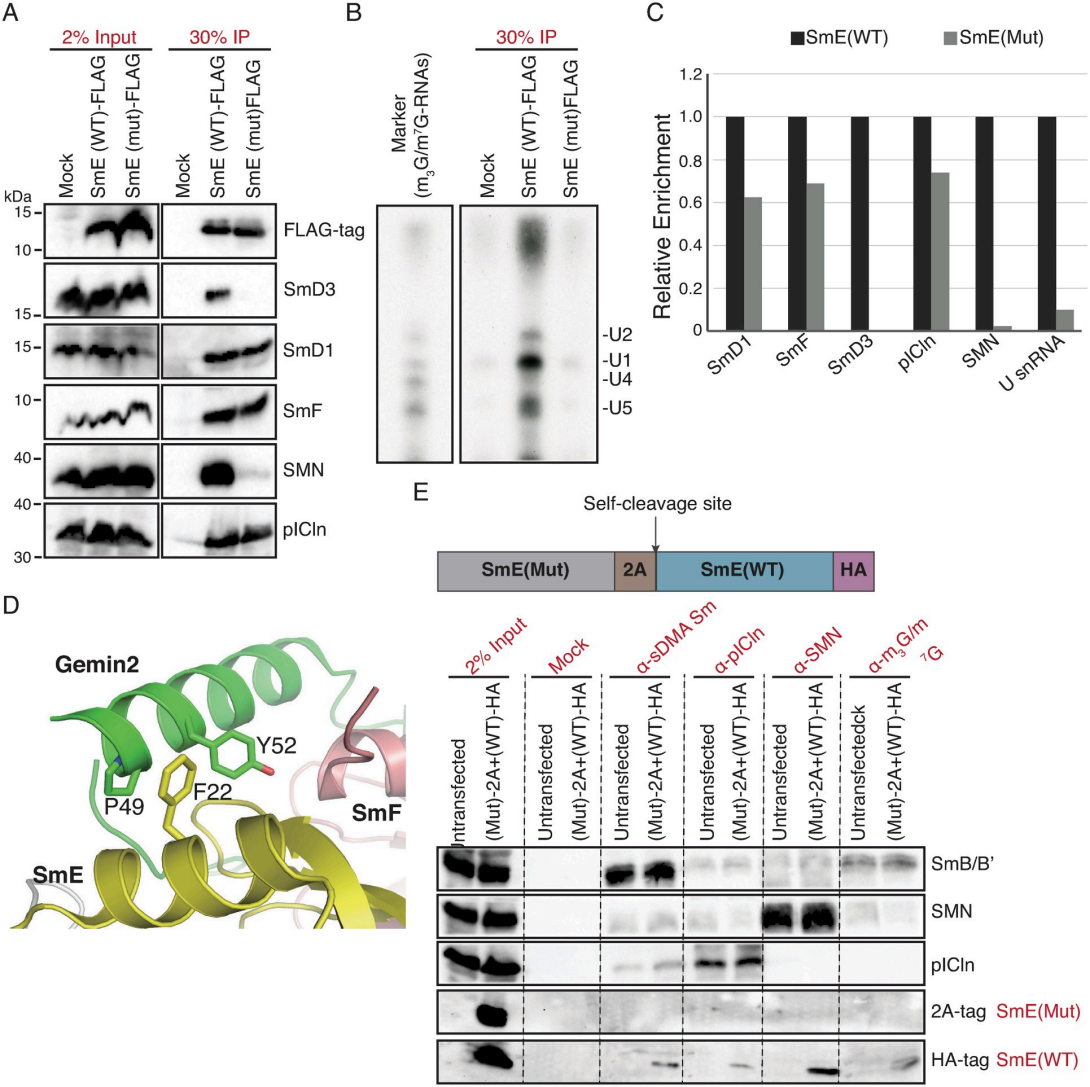
The missense mutation impairs the biogenesis of spliceosomal U snRNPs during Sm core assembly. (A-E), The NSM mutation in SmE impairs its interaction with U snRNP assembly machinery and incorporation into U snRNPs. (A), Anti-FLAG immunoprecipitation after transient transfection in HEK293T cells and western blotting analysis for co-precipitated U snRNP intermediates. Mock immunoprecipitations were performed with untransfected lysates. (B), 3’-end labeling of co-precipitated RNA and autoradiography. RNA immunoprecipitated using the H20 antibody against m_3_G/m^7^G cap of U snRNAs was used as reference. (C), Quantification of the data shown in A and B from two independent biological replicates, with black bars representing wild type and gray bars representing mutant SmE. (D), Predicted structural model for interference of the SmE mutation in its interaction with Gemin2, based on the PDB structure 4V98. (E), Immunoprecipitation using antibodies specific to Sm proteins, SMN, pICn and U snRNA cap, with lysates from HEK293T cells transfected with dual expression plasmid encoding 2A-tagged mutant SmE and HA-tagged wild type SmE and western blotting to analyze the integration of the wild type and mutant SmE into U snRNP biogenesis pathway.

To follow the path of SmE into U snRNPs, FLAG-tagged wild type or mutant proteins were overexpressed in HEK293 cells. The tagged proteins were then immunoprecipitated using anti-FLAG antibodies and co-precipitated factors indicative for defined U snRNP biogenesis intermediates were detected by western blotting (Fig 2A and 2C). Interestingly, no significant change in the interaction of mutant SmE with either SmF, SmD1 or pICln was observed when compared to the wild type protein. This suggests that the pathogenic missense mutation did not interfere with the early phase of U snRNP biogenesis, including formation of SmE/F/G heterooligomer and the 6S complex at the PRMT5 complex. However, only the wild type but not the mutant SmE protein interacted efficiently with SmD3 as well as the SMN complex (Fig 2A and 2C), suggesting that the SmE mutant was defective in the transfer from the PRMT5 complex onto the SMN complex, which is in turn a pre-requisite for the subsequent loading onto U snRNA. In agreement with this notion, 3’-end labeling of the RNA co-precipitated with the SmE-FLAG immunoprecipitations revealed that only wild type SmE was able to efficiently interact with U snRNAs (Fig 2B and 2C). Together these data show that the mutant SmE is unable to be incorporated into U snRNPs (Fig 2B, quantification in Fig 2C).

Since the interaction of mutant SmE with the SMN complex is affected, we used the previously published structure of the 8S U snRNP assembly intermediate (Gemin2-SMNΔC bound to 6S, PDB entry 4V98) [37], to *in silico* model the effect of the mutation. As evident from structural data of Gemin2 in association with Sm proteins, Phe22 of SmE is part of a binding module that interacts with Pro49 and Tyr52 of Gemin2 (Fig 2D). The identified SmE mutation (c.65T>C) changes the polarity of the amino acid residue from hydrophobic (Phe) to hydrophilic (Ser), which is incompatible with the detected mode of interaction.

To recapitulate the disease condition where both wild type and mutant SmE are expressed within the cell, we co-expressed HA-tagged wild type SmE and 2A-tagged mutant SmE in HEK293 cells from a dual expression plasmid and tested how they are processed by the U snRNP assembly pipeline (Fig 2E). The dual expression construct was designed with a post-translational self-cleaving 2A tag between the mutant and wild type SmE (Fig 2E), giving raise to equal amounts of exogenous 2A-tagged mutant and HA-tagged wild type SmE in each transfected cell. We then performed immunoprecipitations using antibodies specific to endogenous U snRNPs (Y12 which predominantly immunoprecipitates U snRNPs and not Sm intermediates), pICln and SMN. As expected, while the wild type SmE was able to efficiently interact with the U snRNP assembly machinery and hence was incorporated into U snRNPs, the mutant was not enriched in any of the immunoprecipitations (note that due to the presence of the highly abundant endogenous Sm protein pool, the efficiency of immunoprecipitation of the tagged proteins was low as compared to those shown in Fig 2A–2C).

**Fig 3:**
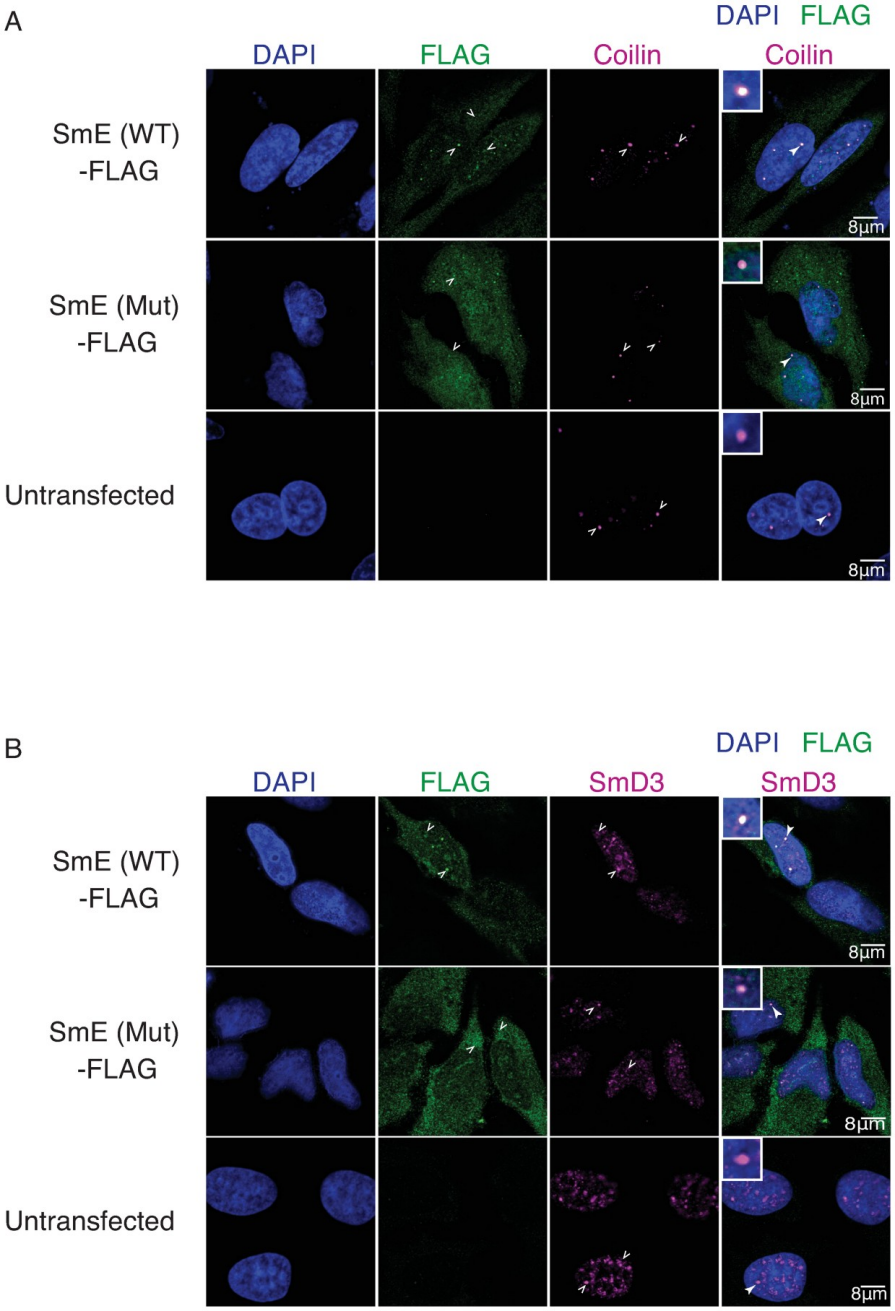
NSM mutation causes mis-localization of the SNRPE/SmE protein. (A-B), Indirect immunofluorescence and confocal microscopy of HeLa cells transfected with either FLAG-tagged wild type or mutant SmE (WT/Mut) or left untransfected (negative control). Empty white arrowheads indicate localization pattern observed and filled white arrowheads indicate zoomed in region shown in the overly inset. (A), Top panel shows clear co-localization of wild type SmE (green) and coilin (magenta) in CBs and middle panel shows most of the mutant SmE (green) distributed in the cytoplasm with a minor fraction in the nucleus and co-localizing with coilin (magenta) in CBs. (B), While wild type SmE (green, top panel) co-localizes with SmD3 (magenta) in CBs and splicing speckles, the mutant SmE (green, middle panel) is predominantly cytoplasmic with marginal co-localization with SmD3 in CBs or in nuclear speckles.

We also performed immunostaining of HeLa cells transiently transfected with either the FLAG-tagged wild type or mutant SmE and studied the co-localization of the exogenously expressed SmE to the CBs (the subnuclear structures for U snRNPs maturation) and to U snRNPs [41]. As expected, the wild type SmE co-localized to CBs as confirmed by a strong co-localization with the CB marker protein coilin (Fig 3A, top panel) and were also efficiently targeted to nuclear speckles as can be seen with co-localization with SmD3 (Fig 3B, top panel). However, in keeping with our immunoprecipitation results, the SmE mutant was localized to the cytoplasm, at times even forming very small foci, or non-specifically dispersed in the nucleus (Fig 3A and 3B, middle panel), showing that the mutant fails to be incorporated into U snRNPs. We conclude that the non-specific nuclear distribution of SmE results from excess of overexpressed exogenous SmE that likely diffuses into the nucleus in the absence of cognate interactors. Together, these results demonstrate that the mutation (c.65T>C(p.Phe22Ser)) in SmE impairs its incorporation into U snRNPs due to its inability to interact with the SMN complex. The early assembly phase, however, appears to be unaffected by this mutation.

### The SNRPE/SmE deficiency results in reduced levels of U snRNPs in patient

Taking into account our biochemical data, we hypothesized that the U snRNP levels in the patient are likely reduced. To this end, we first performed immunostaining and confocal microscopy analysis of control primary fibroblasts and patient fibroblast (S1A and S1B Fig). We found a clear difference in the distribution of U snRNAs (m_3_G/m^7^G cap) in patient cells. While in control fibroblasts U snRNAs were found predominantly within the nuclei (S1A Fig, top panel), there was a marked increase in U snRNAs in the cytoplasm of the patient fibroblasts (S1A Fig, bottom panel). Additionally, levels of Sm proteins in the nuclei of patient fibroblasts was down-regulated (S1A and S1B Fig). CBs are however absent in control as well as patient fibroblasts (S1A and S1B Fig) since CBs are known to be absent in primary cells [42]. Since free U snRNAs that are not assembled into U snRNPs are prone to degradation [43], we proposed that the decrease in U snRNP assembly might result in a reduction in the total U snRNA pool within the patient fibroblasts. We analyzed the U snRNA transcript levels in patient and control fibroblasts using qRT-PCR and the SmE expression level in fibroblasts by RT-qPCR and Western blotting (S1C and S1D Fig). Interestingly, among the U snRNAs tested, we found a clear reduction in the U1 snRNA abundance and a modest decrease in U2 and U4 snRNAs in patient fibroblasts (S1C Fig). We then performed anti-Sm immunoprecipitation from control and patient cells and analyzed the co-precipitated RNA by 3’-end labeling (S1E and S1F Fig). We found a distinct difference in the amount of co-precipitated U snRNAs, with the U1 snRNA levels being the most affected. We conclude that the effects are enhanced specifically in the case of U1 snRNP since the U1–70K protein is known to interact with SMN complex to increase U1 snRNP assembly in cells [44] and thus the strongest effect would be observed for the most abundantly assembled U snRNP.

### The SNRPE/SmE deficiency causes widespread splicing alterations

**Fig 4:**
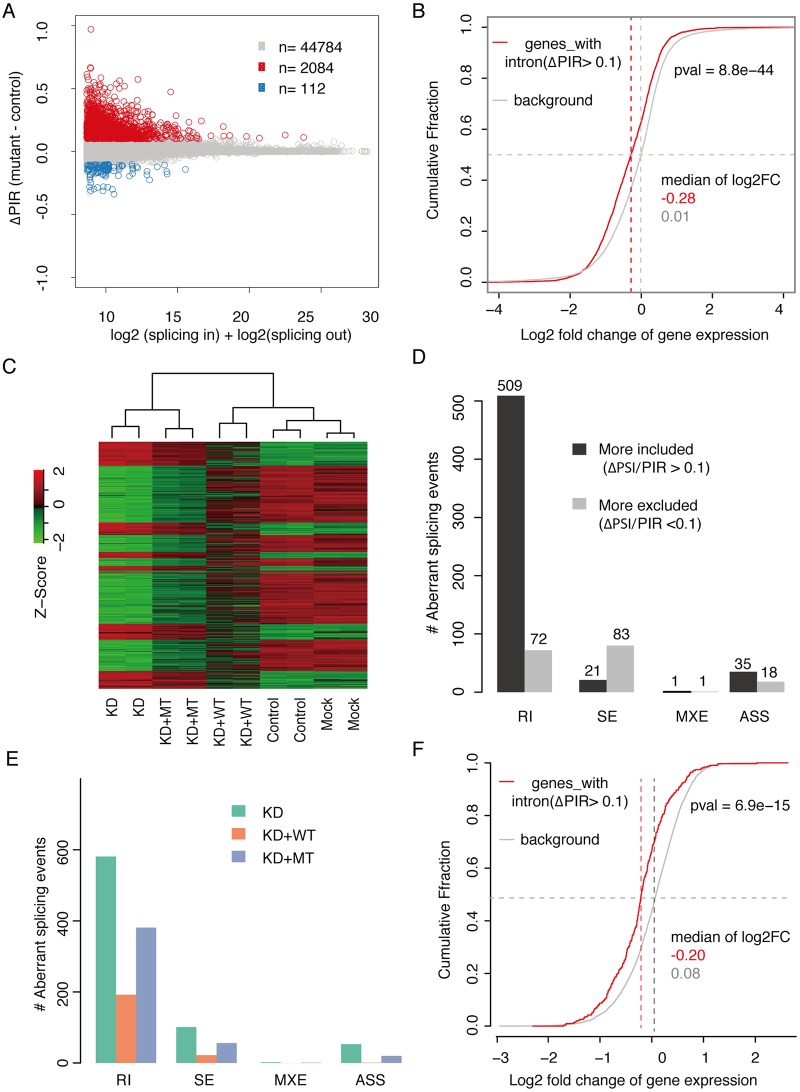
The identified mutation impairs the functionality of SNRPE/SmE in mRNA splicing. (A), The mRNA splicing in patient derived fibroblast cells is impaired. MA plot shows the intron retention was dramatically increased in patient derived fibroblast cells. X axis: the sum of log2 transformed splicing in and splicing out reads number for each intron. Y axis: difference in percentage of intron retention (PIR) between fibroblast derived from the patient (mutant) and healthy control. (B), The intron retention leads to decreased gene expression. Backgrounds are those genes without any intron showing significantly increased retention. (C), Heatmap illustrating expression of 11670 protein coding genes (average RPKM>1) in HEK293 cells among different experimental conditions. Control, control siRNA; KD, SmE siRNA; KD+WT, SmE siRNA+wild type SmE; KD+Mut, SmE siRNA+mutant SmE. (D), Number of aberrant splicing events induced by SNRPE/SmE knockdown (KD) comparing with control. RI, retained intron; SE, skipped exon; MXE, mutually exclusive exon; ASS, alternative splice site. (E), Numbers of aberrant splicing events in KD, KD+Mut and KD+WT comparing to control. (F), The intron retention leads to decreased gene expression. Backgrounds are those genes without any intron showing significant increased retention.

The results above suggest that the identified mutation (c.65T>C (p.Phe22Ser)) in SmE leads to reduced levels of Sm-class snRNPs. As these are the major *trans*-acting factors in pre-mRNA processing, we next asked whether the mutant SmE impacts on the splicing profile of cells. To address this, the RNA was extracted from fibroblast cells derived from the patient and three healthy individuals, and subjected to RNA sequencing. Indeed we observed tremendous altered splicing events between the patient cell and controls, with intron retention (RI) being the most frequently impacted splicing event. As shown in Fig 4A, more than 2084 introns showed significantly increased intron retention (p < 0.001, fdr < 0.05, ΔPercentage of Intron Retention (ΔPIR: mutant—control) > 0.1) in the patient cells while only less than 112 introns showed significant decreased retention (p < 0.001, fdr < 0.05, ΔPIR < -0.1). Intron retention often introduces premature termination codon (PTC) into the affected mRNAs, which triggers nonsense mediated decay (NMD) and potentially also other mRNA decay pathways. We therefore examined the changes in the expression levels of transcripts displaying increased intron retention. Consistent with our assumption, these transcripts show significantly decreased expression between the patient and control comparing to those genes without any introns with increased retention (Mann-Whitney test, p = 8.8e-44) (Fig 4B).

To check whether the splicing defects observed in the patient fibroblast cells could be rescued by the presence of exogenous wild type SmE protein, we exogenously overexpressed wild type SmE in the patient fibroblast cells and performed the RNA-seq. In total, more than 350 million reads were obtained for triplicate experiments and around 93% of them could be uniquely mapped to human reference genome. Given that the splicing defect observed in the patient fibroblast cells was predominantly manifested as increased intron retention, we focused our analysis here on intron retention. By applying the same approach as described above, we firstly compared between the patient fibroblast cells with exogenous wild type SmE and those without. As shown in S2 Fig, after overexpression of wild type SmE (S2A Fig), 2201 introns were less retained while only 414 introns were more retained (S2B Fig). Moreover, when we compared the patient fibroblast cells with exogenous wild type SmE to fibroblast cells from healthy control, as shown in S2C Fig, much less splicing changes were detected and the direction of changes was more symmetric, in contrast to the comparison between the patient and healthy control fibroblast cells (Fig 4A). To further evaluate the rescue efficiency, we plotted the splicing changes in two comparisons, i.e. healthy control vs patient (X axis), and patient with overexpressing SmE vs patient without SmE overexpression (Y axis) (S2D Fig). Among 2084 significant aberrant splicing events that were detected in the patient (Fig 4A), 1130 of them were significantly rescued (S2D Fig). These results, together demonstrated that overexpression of wild type SmE in patient fibroblasts could indeed reduce the predominant splicing defect, i.e. intron retention, observed in the patient fibroblast cells.

To further analyze the functionality of mutant SmE in mRNA splicing and gene expression, the expression level of endogenous SmE was knocked down (KD) by siRNA targeting the 3’ UTR region in HEK293 cell, resulting in reduction of the expression level of SmE by approximately 80% (S3 Fig). Within this background, either wild type or mutant SmE was expressed and RNA was then prepared for mRNA sequencing. In total, more than 30 million high quality reads were obtained for each sample and around 93% of them could be uniquely aligned to the human reference genome (hg19). Among 11670 expressed genes (average RPKM>1), 1060 showed significant alterations in the KD group as compared to the control (BH-adjusted P value < 0.01, |log2 fold change| > 1). Importantly, these dramatic changes in the gene expression profile could be reversed by overexpression of wild type SmE, whereas the mutant was much less effective (Fig 4C). A same pattern was also observed for the alteration of mRNA splicing: the massive aberrant splicing defect caused by SmE deficiency could be dramatically reduced by overexpression of wild type, but not mutant SmE (Fig 4D and 4E). As already observed in the patient-derived fibroblasts, mRNA transcripts with increased intron retention were often down-regulated in KD HEK293 cells. Taken together, these results reveal that the identified mutation impairs the functionality of SmE protein leading to extensive abnormal gene expression and aberrant mRNA splicing.

Furthermore, to examine whether the retained introns, either in the patient fibroblast cells or in HEK293 cells upon SmE knockdown, shared any characteristics, we analyzed 136 features using the method as described by Braunschweig *et al*. [45]. As shown in S4 Fig, the features that are sensitive to SmE dysfunction in both the patient fibroblast cells and HEK293, are quite similar, with the GC content is the most significant one.

### The SmE deficiency disturbs brain development of zebrafish

**Fig 5:**
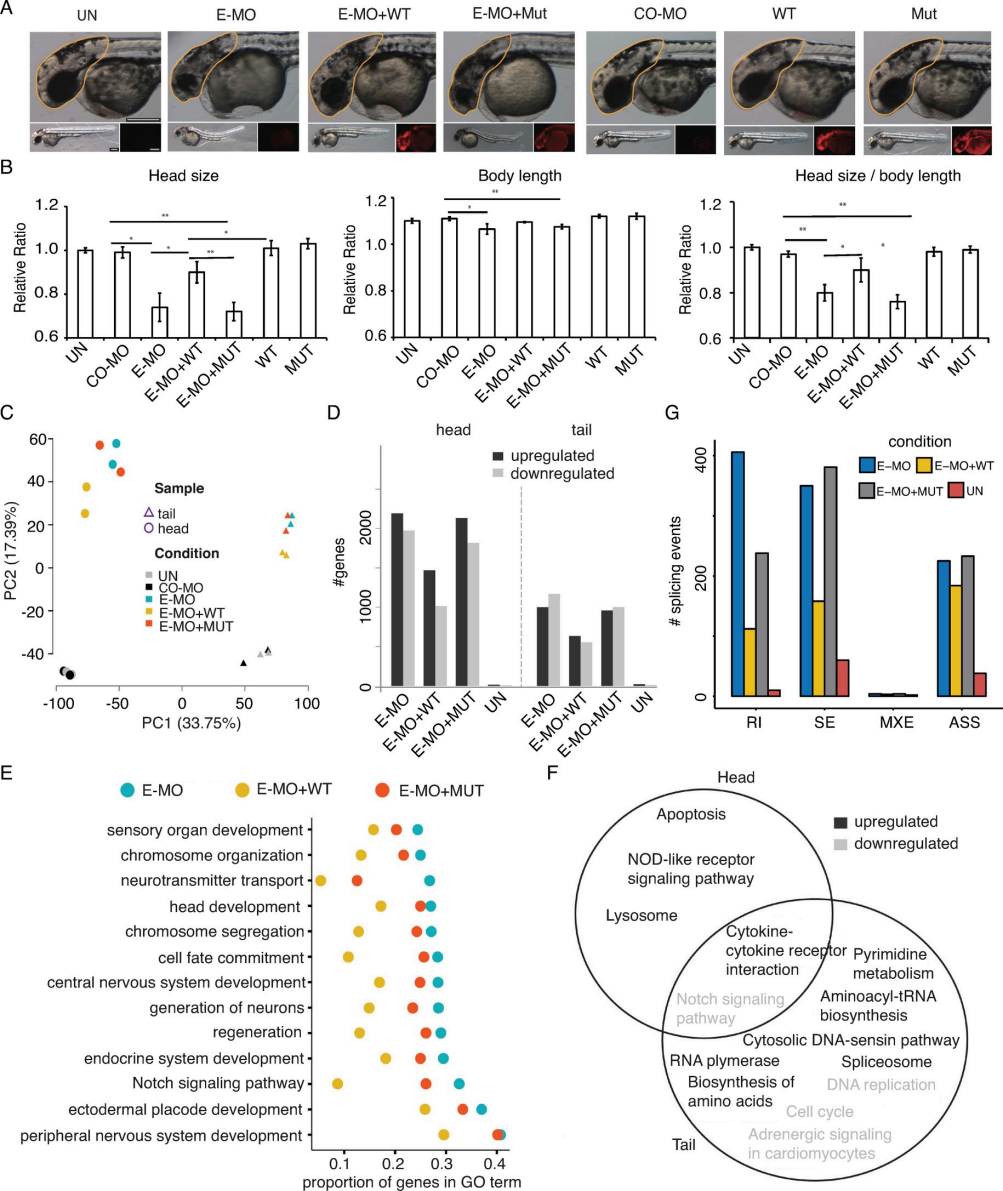
The SNPRE/SmE deficiency interferes with zebrafish brain development. (A), Measurement of zebrafish head size across different experimental conditions. CO-MO, control morpholino; E-MO, SmE morpholino. The morpholino and/or *in vitro* transcribed RNA are injected into embryo at 1-cell stage. Yellow line marked the region for quantification. (B), Quantification of zebrafish head size. Left, head size; middle, body length; right, head size normalized by body length. UN, un-injected; CO-MO, control morpholino; E-MO, SmE morpholino; WT, Wide type SmE gene *in vitro* transcript; Mut, mutant SmE gene *in vitro* transcript; The UN is normalized to 1. * P<0.05; ** P<0.01. (C), PCA analysis of the expression of 16067 protein coding genes (RPKM > 1) in zebrafish head and tail samples under different conditions. (D), Numbers of differentially expressed genes comparing to CO-MO. (E), Proportion of DEGs in 14 significant enriched dysregulated GO terms (biologic process, E-MO versus CO-MO, BH-adjusted p < 0.001). (F), Overlap of enriched dysregulated KEGG pathways (E-MO versus CO-MO, BH-adjusted p < 0.001) between zebrafish head and tail samples. G, Numbers of aberrant splicing events comparing to CO-MO.

To explore the functional consequence of the identified SmE defect *in vivo*, we used zebrafish as a model to dissect the effect of SmE deficiency on animal development. By injecting a morpholino (E-MO) targeting the translation initiation site of zebrafish SmE (zSmE) into fertilized zebrafish embryos at 1-cell stage, the endogenous zSmE levels were decreased after 48h injection (S5 Fig). To analyze the impact of zSmE on head development, the head size of embryos injected with E-MO or a control morpholino (CO-MO) was measured after 48 hours post fertilization. The head size of zebrafish injected with E-MO was significantly decreased (25% reduction) compared to CO-MO injected embryos (Fig 5A and 5B). This phenotype is unlikely to be the consequence of a general developmental delay, since the swim bladder and pigmentation of morphants were phenotypically normal. Although we observed a statistically significant difference in the body length between E-MO and CO-MO, the magnitude of the change is only marginal (Fig 5A and 5B).

To validate that this phenotype is caused by reduced zSmE, rescue experiments were performed. The E-MO was co-injected with *in vitro* transcribed mRNA encoding 2A-mCherry coupled with wild type zSmE (zSmE(WT)-2A-mCherry) lacking the binding site for E-MO. Importantly, the co-injection of E-MO and zSmE(WT)-2A-mCherry could successfully rescue the head-size phenotype. Therefore, the observed phenotype in E-MO injected zebrafish is specifically caused by depletion of zSmE (Fig 5A and 5B). However, co-injection of E-MO and the *in vitro* transcribed mutant zSmE mRNA (zSmE (Mut)-2A-mCherry) failed to rescue the defect (Fig 5A and 5B). Furthermore, overexpression of either wild type or mutant zSmE (WT or Mut)-2A-mCherry alone did not show any phenotype (Fig 5A and 5B). Thus, SmE is required for proper brain development in zebrafish and its deficiency causes a patient-like phenotype.

### Molecular mechanisms underlying zebrafish phenotypic changes induced by SmE deficiency

The results in the patient-derived fibroblasts and in the HEK293 cells revealed that, when carrying the identified mutation, SmE fails to enter the biogenesis pathway of spliceosomal U snRNP, resulting in aberrant mRNA splicing and alteration of the gene expression program. We hence investigated whether the head phenotype in zebrafish is likewise caused by splicing defects culminating in aberrant gene expression patterns. To explore this, RNA from the head and tail regions of untreated zebrafish controls were compared with RNA from the same region isolated from morpholino-injected zebrafish (CO-MO, E-MO alone, and E-MO+WT as well as E-MO+Mut combinations were analyzed). In total, ~680 million reads were obtained and 92.3% of them could be uniquely aligned to the zebrafish reference genome (danRer10).

As expected, zebrafish head and tail have distinct expression profiles as evident from their divergent transcript profiles (Fig 5C). In addition, the overall PCA clusters of embryos injected with E-MO and E-MO+Mut significantly differed from untreated and CO-MO injected samples, while the rescue E-MO+WT represented an intermediate state between these two groups in both head and tail (Fig 5C).

By comparing each fish treatment to the CO-MO control, thousands of differentially expressed genes (DEG) were identified in each comparison (Fig 5D). To test whether these alterations are a direct consequence of zSmE deficiency, we next attempted to rescue the wild type transcriptome by the co-expression of zSmE variants. Indeed, upon co-expression of wild type zSmE the number of DEG was drastically reduced, while DEG numbers in fish co-expressing mutant zSmE was comparable to the zSmE knockdown (Fig 5D). Of note, the number of DEGs in the tail of E-MO zebrafish was much lower than that in the head (Fig 5D), suggesting that the latter was more sensitive to zSmE deficiency.

Consistent with the observed phenotypic changes in zSmE deficient fish, down-regulated DEGs in head are enriched for factors implicated in head development, central nervous system development and cell fate commitment (Fig 5E). Importantly, the proportion of DEGs clustering in these GO terms was dramatically reduced by co-expressing of wild type zSmE but not its pathogenic mutant (Fig 5E). The KEGG pathway analysis showed that the zSmE knockdown affected some pathways such as the Notch signaling pathway in both head and tail (Fig 5F). In contrast, other pathways such as apoptosis were only activated in zebrafish head by E-MO (Fig 5F) and may explain the death of neurons and reduced brain size. Not only alterations in gene expression but also aberrant splicing induced by zSmE deficiency could be rescued by expressing wild type but not mutant zSmE (Fig 5G). Interestingly, the introns more retained due to zSmE deficiency shared similar features as those due to SmE dysfunction in the patient fibroblast and HEK293 cells (S4 Fig). Taken together, these results suggest that the small brain size caused by zSmE deficiency is, likely, a consequence of altered gene expression and aberrant splicing.

### The *EMX2* aberrant splicing is a target of defects in constitutive splicing machinery and causes the microcephaly phenotype

Our RNA-seq data raised the possibility that the phenotype of zSmE deficient zebrafish might be a consequence of disturbed transcription factor networks controlling neuron differentiation as well as apoptosis (Fig 5E and 5F).

**Fig 6:**
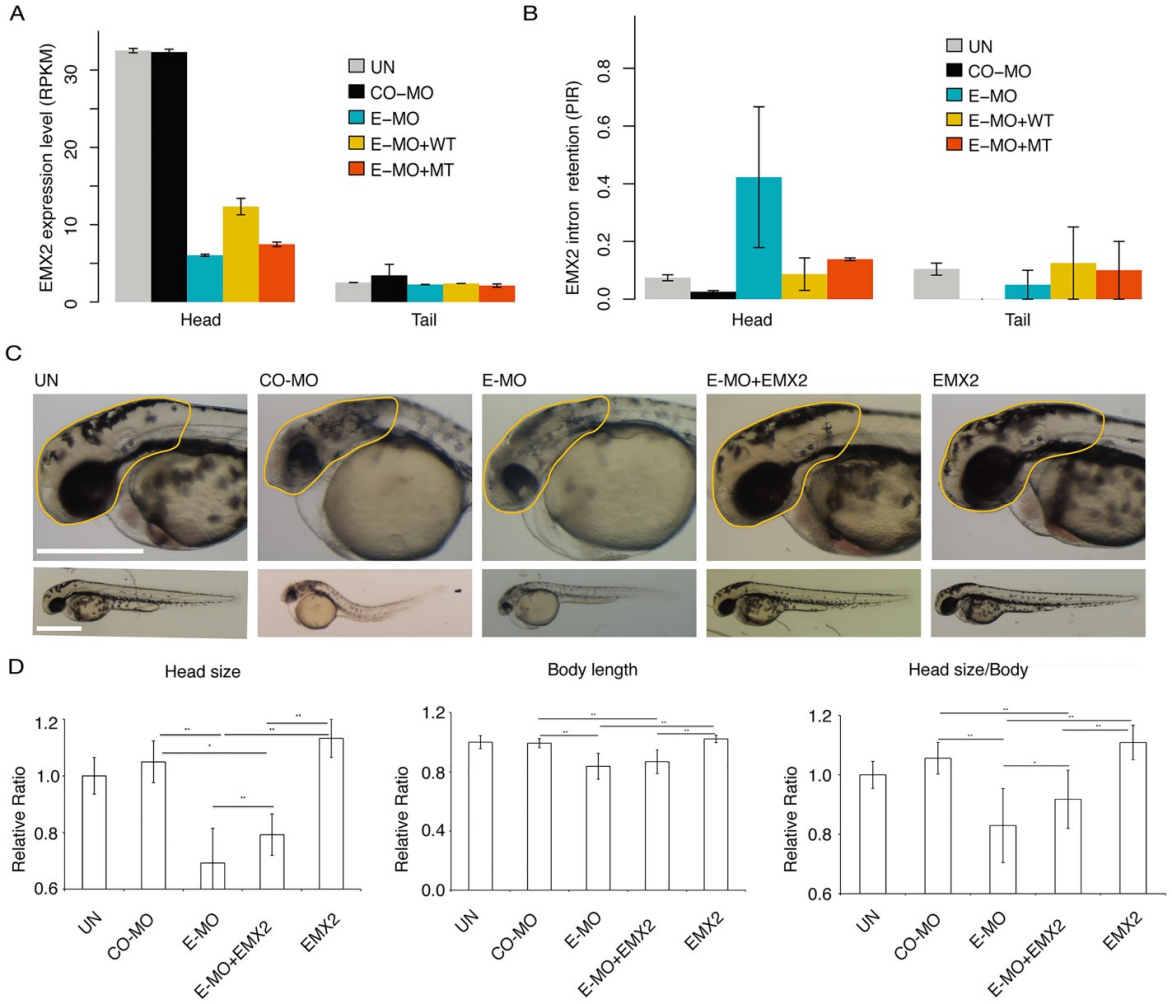
The brain defect caused by SNPRE/SmE deficiency can be partially rescued by transcription factor EMX2. (A), The expression of *EMX2* gene is specifically disturbed in head of zebrafish after zSmE deficiency. (B), The zSmE deficiency leads to increased intron retention of EMX2. (C), The head defect caused by zSmE deficiency can be partially rescued by transcription factor EMX2. The morpholino and/or *in vitro* transcribed RNA is injected into embryo at 1-cell stage. Yellow line marked the region for quantification. (D), Quantification of zebrafish head size. Left, head size; middle, body length; right, head size normalized by body length. UN, un-injected; CO-MO, control morpholino; E-MO, SmE morpholino, EMX2, *EMX2* gene *in vitro* transcript. The UN is normalized to 1. * P<0.05; ** P<0.01.

*EMX* genes (also known as empty spiracles homeobox) are vertebrate cognates of *Drosophila* head gap gene, *empty spiracles* (*ems*). EMX2, a homeobox-containing transcription factor, plays critical roles in controlling patterning and proliferation of dorsal telencephalic progenitors [46,47]. Yoshida *et al*. [48] reported that *Emx2* defective mice lose the dentate gyrus and display greatly reduced hippocampus and medial limbic cortex size. *Emx2* has also been associated with the diseases of schizencephaly [49]. Importantly, our zSmE deficient zebrafish displayed reduced gene expression of the *Emx2* gene (log2 fold change = -2.42, BH-adjusted p = 1.28e-55) and increased intron retention in EMX2 mRNA (ΔPIR = 0.39, p = 9.6e-5, fdr < 0.05) (Fig 6A and 6B).

This effect is strictly dependent on zSmE deficiency, as both intron retention and gene expression change can be partially rescued by WT but less well by mutant zSmE (Fig 6A and 6B). Due to the critical role of EMX2 in controlling patterning and proliferation of dorsal telencephalic progenitors, we explored whether alterations of the EMX2 transcript is causative for the zebrafish phenotype. For this, we tried to rescue the head size phenotype in zSmE depleted zebrafish by co-injection of *in vitro* transcribed EMX2 transcripts. Indeed, the co-injection of EMX2 *in vitro* transcript with E-MO can partially rescue the brain defect (Fig 6C and 6D). Of note, we observed only a partial rescue, which is likely due to the fact that zSmE deficiency also affects the splicing of many other functional relevant genes. Furthermore, application of EMX2 mRNA alone shows no phenotype (Fig 6C and 6D). These results reveal that EMX2, as a downstream target, might act as a key factor as its splicing defects further amplifies the consequence caused by zSmE deficiency.

## Discussion

In higher eukaryotes, the specific morphology and physiological capacities of different cell types is achieved through coordinated precise spatio-temporal expression of lineage specific genes. Alternative splicing (AS), through differential selection of alternative splice sites in pre-mRNA, is not only used to increase the coding capacity of the genome, but also extensively applied to guide the developmental regulation [7]. Defects of mRNA splicing are frequently related to human disease [17,50,51].

Here, we demonstrate that a heterozygous missense mutation (c.65T>C (p.Phe22Ser)) in *SNRPE*/*SmE* gene causes aberrant mRNA splicing and abnormal gene expression, leading to a severe brain defect through SNRPE/SmE deficiency (Figs 4 and 5). Saltzman *et al*. [52] previously showed that the SmB/B’ protein, another basal component of the spliceosome, self-regulates its expression by inclusion of a highly conserved cassette exon to regulate alternative splicing through affecting the availability of spliceosomal U snRNPs. Although the SmE protein is also a basal component of spliceosome, the effect of SNRPE/SmE on mRNA splicing and its physiological role has never been investigated. Our results revealed that, similar to down-regulation of core spliceosomal proteins [53,54], the *SNRPE/SmE* (c.65T>C (p.Phe22Ser)) mutation impairs the biogenesis of spliceosomal U snRNPs (Figs 2 and 3), leading to aberrant mRNA splicing in *in vitro* HEK293 cells (Fig 4) and *in vivo* zebrafish samples (Fig 5). In zebrafish, the specific depletion of endogenous SNPRE/SmE mediated by translation initiation blocking morpholino, leads to decreased head size (Fig 5A and 5B), which successfully recapitulate the patient phenotype. Similar phenomena were also observed in previous studies [53,54]. Bezzi *et al*. [54] showed that conditional knockout of PRMT5 in the central nervous system (CNS) of mice leads to smaller brain, early postnatal death and aberrant mRNA splicing. As a type II arginine methyltransferase [55], PRMT5 acts together with pICln and WDR77/WD40 to symmetrically methylate the arginine residues in SmB/B’, SmD1 and SmD3 proteins to increase their affinity to SMN complex for promoting the spliceosomal U snRNPs assembly [38,56]. Jia *et al*. [53] reported that mutation of a U2 snRNA gene in mice causes the global disruption of alternative splicing and neurodegeneration. In U2 mutant mice, the size of the cerebellum decreases through progressive neuron loss. No matter whether cells face a conditional knockout of PRMT5 or a depletion of U2 snRNA or SNRPE/SmE, the direct consequence is the reduced availability of spliceosomal U snRNPs. The CNS, as the most complex structure, has the highest degree of alternative splicing to keep the diversity of transcriptome and proteome to guide correct developmental fates [57,58]. Therefore, it is reasonable to assume that the CNS is most sensitive to aberrant mRNA splicing and similar phenotypes can be observed under these conditions.

Among the different classes of alternative splicing (AS) events, intron retention (IR) is the least studied and usually regarded as the consequence of mis-splicing. However, an increasing number of studies have shown that regulated IR is widely used as a physiological mechanism to functionally tune the transcriptomes [59–61]. Wong *et al*. [60] showed that, during granulocyte differentiation, IR coupled with NMD is applied as an energetically favorable way to precisely control gene expression. Yap *et al*. [59] demonstrated that IR is applied to coordinated regulation of neuronal steady-state mRNA levels to guide the neuron differentiation. Therefore, aberrant IR can be related to diseases as Bezzi *et al*. [54] and Jia *et al*. [53] reported that the homeostasis of IR is disrupted after PRMT5 depletion or U2 snRNA mutation. IR is also observed as the most abundant aberrant splicing type in the patient-derived fibroblast cells, SNRPE/SmE depleted HEK293 cells and zebrafish zSmE knockdown head samples (Figs 4 and 5). Molecular analysis demonstrates that the extent of aberrant IR is negatively correlated with gene expression, which might be mediated through NMD or nuclear sequestration (Fig 4B and 4F). Further KEGG pathway and GO term analyses of expression modulated genes in zebrafish head with SNRPE/SmE deficiency show that the p53 signaling pathway is enriched in the up-regulated genes whereas the down-regulated genes are significantly enriched in neuron development (Fig 5E and 5F). The up-regulation of p53 signaling pathway was also reported by Jia *et al*. [53] and Bezzi *et al*. [54] and considered to contribute to neuronal death. Therefore, like with the PRMT5 depletion or U2 snRNA mutation, the p53 signaling pathway activation might contribute similarly to the SNRPE/SmE deficiency phenotype.

Among those down-regulated genes related to neuron differentiation and brain development, LHX5 promotes the forebrain development through inhibiting Wnt signaling [62]. LHX2 and LHX9 guide the neuronal differentiation and compartmentalization in the caudal forebrain through regulating Wnt signaling [63]. EMX2 functions in the development of dorsal telencephalon, the EMX2 mutant shows defect of dentate gyrus and significantly reduced size of the hippocampus and medial limbic cortex [48,64]. Due to the phenotype similarity between EMX2 mutant and SNRPE/SmE mutant, it is tempting to speculate that the phenotype of SNRPE/SmE mutant might be mediated through disrupting the expression of transcription factors responsible for early brain development, such as EMX2. The result that injection of an *in vitro* generated transcript encoding EMX2 can partially rescue the phenotype of reduced SNRPE/SmE (Fig 6), is consistent with this hypothesis. The data are consistent with the idea that during early development, the SNRPE/SmE deficiency disturbs the brain development through interfering with the splicing of transcription factors, which are responsible for guiding the early brain development.

In addition to the mutation we reported in this study, Pasternack *et al*. demonstrated that the mutations of SNRPE/SmE (c.1A>G (p.M1?) and c.133G>A (p.G45S)) can cause the autosomal-dominant hypotrichosis simplex [31]. These mutations affect the solubility of proteins, however, the soluble part can still efficiently integrate into functional spliceosomal U snRNPs. Moreover, Weiss *et al*. identified a dominant mutation (c.153T>A (p.E51D)) in SmE from a hypogonadism mouse strain [65]. Due to the different position of mutations, the effect of mutations on the functionality of SNRPE might be very different. As the basal component of spliceosomal U snRNPs, the consequence of such different effects from different mutations could be further magnified through altered mRNA splicing and stability, especially the splicing/expression of different transcription factors.

Finally, although we identified the *SNRPE* mutation (c.65T>C (p.Phe22Ser)) from only one patient, the biochemical and zebrafish data provide strong evidence to link this mutation to the microcephaly phenotype manifested in this patient. Therefore, this study expands on our understanding of the effects of core spliceosomal machinery defects on early brain development, and provides insight into the etiology of microcephaly.

## Material and methods

### Ethics statement

The study and use of human samples were approved by the Charité Ethics Committee (EA1/212/08), and the patient’s parents provided written informed consent. For the animal research, all experiments in the manuscript were performed with embryos of less than 5 days of age. According to German and EU rule, those experiments need to only to be approved by the local government and not considered to be animal experiments that need special permission. Zebrafish (*Danio rerio*) were bred and maintained as preciously established [66]. All experimental procedures were performed according to the guidelines of the German animal welfare law and approved by the local government (Government of Lower Franconia; Tierschugtzgesetz §11, Abs. 1, Nr. 1 husbandry permit number 568/300-1870/13). All zebrafish experiments have been performed at embryonic stage prior to independent feeding. Used zebrafish strains: TL (*Tüpfel long fin; leo*^*t1*^*/lof*^*dt2*^; ZFIN ID: ZDB-GENO-990623-2).

### Exome sequencing

All family members were subjected to exome sequencing. In brief, DNA was extracted from the patient and parents’ blood samples. According to the manufacture’s protocol, the genomic DNA was enriched by Agilent Human All Exon V4 Kit (Agilent Technologies, Santa Clara, CA, USA). The whole exome libraries were subjected to Illumina HiSeq2000 system for 100 cycles single end sequencing. After sequencing, the data analysis for exome sequencing was performed as described before by Fröhler *et al*. [67].

### Cell lines and antibodies

Fibroblasts from the forearm of the patient and age-matched control were established according to a standard protocol and cultured in DMEM with 4.5g/l D-glucose and pyruvate (Invitrogen, Darmstadt, Germany) supplemented with 15% fetal bovine serum (FBS) and 1% penicillin-streptomycin.

SmE lentiviral overexpression plasmid was constructed by replacing the Cas9 cassette on lentiCas9-Blast (Addgene, #52962) with the SmE sequence followed by HA-tag, T2A and mCherry cassettes. For each virus package, HEK293T cells (3×10^5^) were seeded in one well of 6-well plate, and were transfected with plasmid after 24 hours. For the transfection, 10.5ul PEI (1μg/μl, Polysciences, #23966–2) and 3.5μg total plasmid (1μg lentiviral plasmid, 1.5μg pMD2.G (Addgene, #12259), and 1μg psPAX2 (Addgene, #12260) were added to the 200μl Opti-MEM (Thermo, #31985075). After 20 minutes, the mix was added to the cells. 12 hours after transfection, the medium was replaced by the fresh medium. After 48 hours, the supernatant were collected, and clarified by centrifugation (2000g), and filtrated through a 0.45μm filter (Millex, #SLHV033RB). The transduction was done by incubating the viral particles containing supernatant with the patient fibroblast cells overnight in the presence of polybrene (8 μg/μl, Sigma, H9268).

Stable HEK293 T-Rex Flp-In cell lines, inducibly expressing the HA-tagged wild type or mutant SmE protein were constructed and maintained as previously described [68]. For transient transfection, HeLa and HEK293T cells were cultured in DMEM media supplemented with 10% FBS.

The following antibodies were used in this study: anti-SMN (clone 7B10; purified from hybridoma supernatant) [69], rabbit anti-pICln [36], mouse anti-m_3_G/m^7^G cap (H-20, a kind gift from Prof. R. Lührmann) [70], mouse anti-Sm (Y12, a kind gift from Prof. J.A. Steitz) [71], rabbit anti-coilin (H-300, Santa Cruz Biotechnology, sc-32860), rabbit anti-SmD3 (Pierce, PA5–26288), rabbit anti-SmD1 (Pierce, PA5–12459), rabbit anti-SmF (Abcam, ab66895), mouse anti-FLAG (Sigma, F1804 and F3165) and anti-HA (Covance). For western blotting, we used secondary goat antibodies conjugated with horse raddish peroxidase; anti-mouse (Sigma, A4416) and anti-rabbit (Sigma, A6154). For indirect immunostaining we used Cy5-conjugated goat secondary antibody (red channel), anti-rabbit IgG (Jackson ImmunoResearch Laboratories, 111-175-144) and Alexa488-conjugated goat secondary antibody (green channel), anti-mouse (Thermo Scientific, A11017).

### Immunoprecipitation (IP) of proteins and RNA-protein complexes from stable cell lines or transient transfections, 3’-end labeling of RNA

HEK293T cells were seeded in 150mm cell culture dishes and transfected at 80% confluency using Mirus Transit-X2 system as per manufacturer’s protocol for immunoprecipitations with 20μg of SmE wild type or mutant construct or dual-expression plasmid or left untransfected for mock immunoprecipitations. Lysate were prepared 48 hours after transient transfection or after 24 hours of induction of stable cell lines with 100ng/ml doxycycline.

All IP experiments were performed as previously described [43]. Briefly, the cells were homogenized in lysis buffer (50mM HEPES pH7.5, 150mM NaCl, 2.5mM MgCl_2_, 1% NP-40 substitute, RNasin and proteinase inhibitors) and insoluble debris was removed by centrifugation. The supernatant was then collected, concentration estimated using Bradford assay and incubated with Protein-G Dynabeads (Thermo Scientific) coupled with corresponding antibodies or with anti-FLAG agarose M2 affinity gel (Sigma) for 3h at 4°C. After incubation, the beads were washed three times with ice-cold wash buffer (50mM HEPES pH7.5, 300mM NaCl, 2.5mM MgCl_2_) and once with 1×PBS with 2.5mM MgCl_2_. The immunoprecipitate was subsequently dissociated from the beads using 1×Lämmli SDS dye, separated on a SDS-PAGE and analyzed by western blotting or directly treated with TRIzol (Thermo Scientific) for RNA extractions as per manufacturer’s protocol. The precipitated RNA was resuspended in nuclease free water and incubated with ^32^P-pCp and T4 RNA ligase in an overnight reaction at 4°C. The RNA was precipitated after Phenol-chloroform extraction and separated on 8% polyacrylamide-Urea denaturing gel and exposed for autoradiography.

### Immunostaining and confocal microcopy

For immunostaining, HeLa cells were grown on coverslips and transfected with FLAG-tagged wild type or mutant SmE constructs respectively at 70% confluency using Mirus Transit-X2 or left un-transfected (control). After 48 hours of transfection, the coverslips were processed for immunostaining. Control primary human fibroblasts and patient fibroblast were seeded on coverslips and grown to 70% confluency before immunostaining. The cells were washed and fixed with 4% para-formaldehyde and permeabilized with 0.2% Triton X-100 in 1×PBS and blocked with 10% FCS. Primary and secondary antibodies were diluted in 2% FBS. After primary and secondary antibody binding and washes, the coverslips were mounted using Mowiol 4–88 mounting medium. Confocal imaging was carried out using Leica SP5 confocal microscope with photomultiplier and the images were processed using ImageJ software.

### Injection and analysis of zebrafish embryos

The zebrafish (*Danio rerio*) embryos were maintained and harvested as previous described [66]. The translation-blocking morpholino against zebrafish SmE was designed and obtained from Gene tools (SmE MO: 5’-TGTCCTTGTCCTCTGTACGCCATTC-3’) targeting the translation initiation site. Control morpholino was a scrambled nucleotide sequence provided by Gene tools (5’-TGTCGTTCTGCTCTCTACCCCATTC -3’). 1nl of morpholino solution (final concentration 20nM) was injected into zebrafish embryos at the 1–2 cell stage. For RNA rescue and over-expression experiments, *in vitro* transcribed RNA (final concentration of 150pg) encoding the CDS of zebrafish SmE with/without point mutation was fused with mCherry and separated from each other by 2A-tag. To avoid the targeting by SmE morpholino, synonymous codons were used to substitute the 4^th^-7^th^ amino acid positions. The coding sequence was changed from AGAGGACAAGGA to CGTGGCCAGGGT. To quantify the phenotype, the images of embryos were taken at 48 hours post fertilization (hpf), and the size of the heads and length of the body were quantified. All experiments were repeated for three times and the significance of the morphant phenotype was determined by Student’s *t*-test.

### RNA sequencing

Total RNAs were extracted from the patient derived fibroblast cells, HEK293 cell lines, zebrafish heads and tails using TRIzol reagent (Life Technologies) following manufacturer’s instruction. Stranded mRNA sequencing libraries were prepared with 500 ng total RNA according to manufacturer’s protocol (Illumina). The libraries were subjected to Illumina HiSeq 2000 system for 100 cycles single end sequencing.

### RNA-seq data analysis

All RNA-seq reads were aligned to a reference genome (human: hg19; zebrafish: danRer10) by using STAR with transcriptome annotation (human: Gencode v18; zebrafish: ensemble 82). HTseq-Count was further utilized to calculate gene expression by counting uniquely mapped reads within each gene. DEseq2 was then applied to identify differentially expressed genes between different conditions. Based on transcriptome annotation, splicing events including alternative splicing sites (ASS), skipped exon (SE), retained intron (RI) and mutually exclusive exons (MXE) were constructed. Especially for SE and RI, all middle exons and introns were considered potentially to be skipped or retained. Using reads aligned to exon-exon junction and exon-intron boundaries, expression of each splicing event was quantified and further compared between each two different conditions. We used a rank-product based method as described in a previous study [67], to estimate significance (p < 0.001, fdr < 0.05 were defined as significant) by checking consistence among different biological replicates. For zebrafish RNA-seq data analysis, we examined GO and KEGG pathway enrichment (BH-adjusted P value < 0.001) for genes, which were differentially expressed (BH-adjusted P value < 0.001, |log2 fold change| > 2, RPKM > 1) between E-MO and Control-MO, using WEB-based Gene SeT AnaLysis Toolkit (WebGestalt). In brief, we estimated significance of the overrepresentation of up and down regulated genes in each GO term and KEGG pathway, comparing with background genes respectively (all expressed genes, i.e. RPKM > 1). Next, in each significant enriched GO-term, proportions of differentially expressed genes among all genes in the GO term across different comparisons, including E-MO versus Control-MO, E-MO+WT versus Control-MO, and E-MO+MT versus Control-MO, were estimated separately. For enriched KEGG pathways, we also checked the overlap between the results from head and tail RNA-seq data.

## Supporting information

S1 Text Support information-Clinical information of the patient. (DOCX)

S1 Fig U snRNP levels are reduced in the patient due to the SmE mutation. (A-B), Indirect immunofluorescence and confocal microscopy of control and patient fibroblasts. Empty white arrowheads indicate localization pattern observed and filled white arrowheads indicate zoomed in region shown in the overlay inset. (A), Co-staining with DAPI (blue), m_3_G/m^7^G cap of U snRNA (green) and SmD1 (magenta). Control fibroblasts (top panel) show abundant U snRNPs in nuclear speckles and both SmD1 and U snRNAs are predominantly absent from the cytoplasm. In patient fibroblasts (bottom panel) though there is an excellent co-localizaiton of U snRNAs and SmD1, there is a decrease in their nuclear abundance and there is an increase in their cytoplasmic localization. (B), Indirect immunofluorescence and confocal microscopy of DAPI (blue), symmetrically dimethylated (sDMA)-Sm proteins (green) and coilin (magenta). In comparison to the control fibroblasts (top panel), the patient cells (bottom panel) have reduced Sm proteins in the nucleus and an increased cytoplasmic retention. Coilin foci is not present in the images as primary cells lacks CBs. (C), Quantitative real-time PCR analysis of snRNAs and SmE in control (black bars) and patient (gray bars) fibroblasts from two independent biological replicates. (D), The SmE protein expression level in patient and control fibroblasts was checked by western blotting. The tubulin was used as loading control. (E), Immunoprecipitation of Sm proteins from control and patient fibroblasts (bottom panel, western blotting) and autoradiography (top panel) after 3’-end labeling of coprecipitated RNA. Mock indicates immunoprecipitation control without any antibody coupled to the beads. (F), Quantification of autoradiography in E; control in black and patient in gray, from two independent biological replicates.
(TIF)

S2 Fig The impaired mRNA splicing in patient fibroblasts can be rescued by overexpression wild type SmE protein. (A), Wild type SmE protein was successfully overexpressed in the patient fibroblast cells. The expression level was estimated based on RNA-seq data. (B), The MA plot compares the intron retention in the patient fibroblast cells with to those without overexpression of wild type SmE protein; X axis, log2 transformed the product of splicing in and splicing out reads number for each intron; Y axis, difference in percentage of intron retention (PIR) between the patient fibroblast cells with overexpression of wild type SmE protein (OE) and those without (mutant). (C), The MA plot compares the intron retention between the patient fibroblast cells with overexpression of wild type SmE to fibroblast cells from healthy control (control). (D), The scatter plot illustrates the PIR changes between healthy control vs mutant (X axis) and OE vs mutant (Y axis).
(TIF)

S3 Fig The endogenous SmE can be successfully knocked down by siRNA. Western blot analysis shows that the endogenous SmE can be specifically depleted by SmE siRNA, targeting to the 3’ UTR region, and the exogenous HA-tagged SmE protein can be efficiently induced. The β-tubulin is used as loading control.
(TIF)

S4 Fig The KS-statistics for the 18 most representative features among 136 features across different comparisons. The features were compared between group 1 and group 2 (left panel); between group 3 and group 4 (middle panel); between group 5 and group 6 (right panel). The GC content is the most significantly enriched feature among all the three comparisons. Group 1: introns with increased retention in the patient fibroblast cells vs healthy control fibroblast cells (adjusted p < 0.05, delta PIR > 0.1); Group 2: introns without increased retention in the patient fibroblast cells vs healthy control fibroblast (delta PIR < 0.05, p > 0.05), this group serves as background for group 1; Group 3: introns with increased retention in HEK293 upon SmE knockdown vs control HEK293 (adjusted p < 0.05, delta PIR > 0.1); Group 4: introns without increased retention in HEK293 upon SmE knockdown vs control HEK293 (delta PIR < 0.05, p > 0.05), this group serves as background for group 3; Group 5: introns with increased retention in zebrafish upon SmE knockdown vs control (adjusted p < 0.05, delta PIR > 0.1); Group 6: introns without increased retention in zebrafish upon SmE knockdown vs control (delta PIR < 0.05, p > 0.05), this group serves as background for group 5.
(TIF)

S5 Fig The endogenous SmE in zebrafish can be successfully knocked down by SmE morpholino. Western blot analysis shows that the endogenous zSmE can be specifically depleted by SmE morpholino, targeting to the translation initiation site. The β-tubulin is used as loading control. UN, un-injection; CO-MO, control morpholino; E-MO, SmE morpholino.
(TIF)

## References

[1] Berget SM, Moore C, Sharp PA. Spliced segments at the 5' terminus of adenovirus 2 late mRNA. PNAS. 1977; 74: 3171–3175. 10.1073/pnas.74.8.3171 269380 PMC431482

[2] Chow LT, Gelinas RE, Broker TR, Roberts RJ. An amazing sequence arrangement at the 5' ends of adenovirus 2 messenger RNA. Cell. 1977; 12(1): 1–8. 10.1016/0092-8674(77)90180-5 902310

[3] Lerner MR, Boyle JA, Mount SM, Wolin SL, Steitz JA. Are snRNPs involved in splicing? Nature. 1980; 283(5743): 220–224. 10.1038/283220a0 7350545

[4] Wahl MC, Will CL, Lührmann R. The Spliceosome: design principles of a dynamic RNP machine. Cell. 2009; 136(4): 701–718. 10.1016/j.cell.2009.02.009 19239890

[5] Wickramasinghe VO, Gonzàlez-Porta M, Perera D, Bartolozzi AR, Sibley CR, Hallegger M, et al Regulation of constitutive and alternative mRNA splicing across the human transcriptome by PRPF8 is determined by 5′ splice site strength. Genome Biol. 2015; 16(201): 1–21. 26392272 10.1186/s13059-015-0749-3 PMC4578845

[6] Pan Q, Shai O, Lee LJ, Frey BJ, Blencowe BJ. Deep surveying of alternative splicing complexity in the human transcriptome by high-throughput sequencing. Nat Genet. 2008; 40(12): 1413–1415. 10.1038/ng.259 18978789

[7] Nilsen TW, Graveley BR. Expansion of the eukaryotic proteome by alternative splicing. Nature. 2010; 463(7280): 457–463. 10.1038/nature08909 20110989 PMC3443858

[8] Blencowe BJ. Alternative splicing: new insights from global analyses. Cell. 2006; 126(1): 37–47. 10.1016/j.cell.2006.06.023 16839875

[9] Wang ET, Sandberg R, Luo S, Khrebtukova I, Zhang L, Mayr C, et al Alternative isoform regulation in human tissue transcriptomes. Nature. 2008; 456(7221): 470–476. 10.1038/nature07509 18978772 PMC2593745

[10] Witten JT, Ule J. Understanding splicing regulation through RNA splicing maps. Trends Genet. 2011; 27(3): 89–97. 10.1016/j.tig.2010.12.001 21232811 PMC3165201

[11] Barash Y, Calarco JA, Gao W, Pan Q, Wang X, Shai O, et al Deciphering the splicing code. Nature. 2010; 465(7294): 53–59. 10.1038/nature09000 20445623

[12] Boutz PL, Stoilov P, Li Q, Lin C-H, Chawla G, Ostrow K, et al A post-transcriptional regulatory switch in polypyrimidine tract-binding proteins reprograms alternative splicing in developing neurons. Genes Dev. 2007; 21(13): 1636–1652. 10.1101/gad.1558107 17606642 PMC1899473

[13] Graveley BR, Brooks AN, Carlson JW, Duff MO, Landolin JM, Yang L, et al The developmental transcriptome of Drosophila melanogaster. Nature. 2011; 471(7339): 473–479. 10.1038/nature09715 21179090 PMC3075879

[14] Baker BS. Sex in flies: the splice of life. Nature. 1989; 340(6234): 521–524. 10.1038/340521a0 2505080

[15] Xie J, Black DL. A CaMK IV responsive RNA element mediates depolarization-induced alternative splicing of ion channels. Nature. 2001; 410(6831): 936–939. 10.1038/35073593 11309619

[16] Singh RK, Cooper TA. Pre-mRNA splicing in disease and therapeutics. Trends Mol Med. 2012; 18: 472–482. 10.1016/j.molmed.2012.06.006 22819011 PMC3411911

[17] Cooper TA, Wan L, Dreyfuss G. RNA and Disease. Cell. 2009; 136(4): 777–793. 10.1016/j.cell.2009.02.011 19239895 PMC2866189

[18] Niblock M, Gallo J-M. Tau alternative splicing in familial and sporadic tauopathies. Biochemical Soc Trans. 2012; 40(4): 677–680. 10.1042/BST20120091 22817715

[19] Comitato A, Spampanato C, Chakarova C, Sanges D, Bhattacharya SS, Marigo V. Mutations in splicing factor PRPF3, causing retinal degeneration, form detrimental aggregates in photoreceptor cells. Hum Mol Genet. 2007; 16(14): 1699–1707. 10.1093/hmg/ddm118 17517693

[20] Linder B, Hirmer A, Gal A, Rüther K, Bolz HJ, Winkler C, et al Identification of a PRPF4 loss-of-function variant that abrogates U4/U6.U5 Tri-snRNP integration and is associated with retinitis pigmentosa. PLoS One. 2014; 9(11): e111754 10.1371/journal.pone.0111754 25383878 PMC4226509

[21] Tanackovic G, Ransijn A, Ayuso C, Harper S, Berson Eliot L, Rivolta C. A missense mutation in PRPF6 causes impairment of pre-mRNA splicing and autosomal-dominant retinitis pigmentosa. Am J Hum Genet. 2011; 88(5): 643–649. 10.1016/j.ajhg.2011.04.008 21549338 PMC3146730

[22] Maubaret CG, Vaclavik V, Mukhopadhyay R, Waseem NH, Churchill A, Holder GE, et al Autosomal dominant retinitis pigmentosa with intrafamilial variability and incomplete penetrance in two families carrying mutations in PRPF8. Invest Ophthalmol Vis Sci. 2011; 52: 9304–9309. 10.1167/iovs.11-8372 22039234

[23] Venturini G, Rose AM, Shah AZ, Bhattacharya SS, Rivolta C. CNOT3 Is a modifier of PRPF31 mutations in retinitis pigmentosa with incomplete penetrance. PLoS Genet. 2012; 8(11): e1003040 10.1371/journal.pgen.1003040 23144630 PMC3493449

[24] Linder B, Dill H, Hirmer A, Brocher J, GLee GP, Mathavan S, et al Systemic splicing factor deficiency causes tissue-specific defects: a zebrafish model for retinitis pigmentosa. Hum Mol Genet. 2011; 20(2): 368–377. 10.1093/hmg/ddq473 21051334

[25] Cvačková Z, Matějů D, Staněk D. Retinitis pigmentosa mutations of SNRNP200 enhance cryptic splice-site recognition. Hum Mutat. 2014; 35(3): 308–317. 10.1002/humu.22481 24302620

[26] Mordes D, Luo X, Kar A, Kuo D, Xu L, Fushimi K, et al Pre-mRNA splicing and retinitis pigmentosa. Mol Vis. 2006; 12: 1259–1271. 17110909 PMC2683577

[27] Lefebvre S, Bürglen L, Reboullet S, Clermont O, Burlet P, Viollet L, et al Identification and characterization of a spinal muscular atrophy-determining gene. Cell. 1995; 80(1): 155–165. 10.1016/0092-8674(95)90460-3 7813012

[28] Zhang Z, Lotti F, Dittmar K, Younis I, Wan L, Kasim M, et al SMN deficiency causes tissue-specific perturbations in the repertoire of snRNAs and widespread defects in splicing. Cell. 2008; 133(4): 585–600. 10.1016/j.cell.2008.03.031 18485868 PMC2446403

[29] Lynch DC, Revil T, Schwartzentruber J, Bhoj EJ, Innes AM, Lamont RE, et al Disrupted auto-regulation of the spliceosomal gene SNRPB causes cerebro–costo–mandibular syndrome. Nat Commun. 2014; 5: 4483–4488. 10.1038/ncomms5483 25047197 PMC4109005

[30] Bacrot S, Doyard M, Huber C, Alibeu O, Feldhahn N, Lehalle D, et al Mutations in SNRPB, encoding components of the core splicing machinery, cause cerebro-costo-mandibular syndrome. Hum Mut. 2015; 36(2): 187–190. 10.1002/humu.22729 25504470

[31] Pasternack Sandra M, Refke M, Paknia E, Hennies Hans C, Franz T, Schafer N, et al Mutations in SNRPE, which encodes a core protein of the spliceosome, cause autosomal-dominant hypotrichosis simplex. Am J Hum Genet. 2013; 92(1): 81–87. 10.1016/j.ajhg.2012.10.022 23246290 PMC3542472

[32] Edery P, Marcaillou C, Sahbatou M, Labalme A, Chastang J, Touraine R, et al Association of TALS developmental disorder with defect in minor splicing component U4atac snRNA. Science. 2011; 332(6026): 240–243. 10.1126/science.1202205 21474761

[33] He H, Liyanarachchi S, Akagi K, Nagy R, Li J, Dietrich RC, et al Mutations in U4atac snRNA, a component of the minor spliceosome, in the developmental disorder MOPD I. Science. 2011; 332(6026): 238–240. 10.1126/science.1200587 21474760 PMC3380448

[34] Neiswanger K, Stanford DR, Sparkes RS, Nishimura D, Mohandas T, Klisak I, et al Assignment of the gene for the small nuclear ribonucleoprotein E (SNRPE) to human chromosome 1q25–q43. Genomics. 1990; 7(4): 503–508. 10.1016/0888-7543(90)90192-w 2143747

[35] Raker VA, Plessel G, Lührmann R. The snRNP core assembly pathway: identification of stable core protein heteromeric complexes and an snRNP subcore particle in vitro. EMBO J. 1996; 15(9): 2256–2269. 8641291 PMC450151

[36] Chari A, Golas MM, Klingenhäger M, Neuenkirchen N, Sander B, Englbrecht C, et al An assembly chaperone collaborates with the SMN complex to generate spliceosomal snRNPs. Cell. 2008; 135(3): 497–509. 10.1016/j.cell.2008.09.020 18984161

[37] Grimm C, Chari A, Pelz J-P, Kuper J, Kisker C, Diederichs K, et al Structural basis of assembly chaperone- mediated snRNP formation. Mol Cell. 2013; 49(4): 692–703. 10.1016/j.molcel.2012.12.009 23333303

[38] Meister G, Eggert C, Bühler D, Brahms H, Kambach C, Fischer U. Methylation of Sm proteins by a complex containing PRMT5 and the putative U snRNP assembly factor pICln. Curr Biol. 2001; 11(24): 1990–1994. 10.1016/s0960-9822(01)00592-9 11747828

[39] Fischer U, Luhrmann R. (1990) An essential signaling role for the m3G cap in the transport of U1 snRNP to the nucleus. Science. 1990; 249(4970): 786–790. 10.1126/science.2143847 2143847

[40] Mouaikel J, Verheggen C, Bertrand E, Tazi J, Bordonné R. Hypermethylation of the cap structure of both yeast snRNAs and snoRNAs requires a conserved methyltransferase that is localized to the nucleolus. Mol Cell. 2002; 9(4): 891–901. 10.1016/s1097-2765(02)00484-7 11983179

[41] Lemm I, Girard C, Kuhn AN, Watkins NJ, Schneider M, Bordonne R, et al Ongoing U snRNP biogenesis is required for the integrity of cajal bodies. Mol Biol Cell. 2006; 17(7): 3221–3231. 10.1091/mbc.E06-03-0247 16687569 PMC1483051

[42] Hearst SM, Gilder AS, Negi SS, Davis MD, George EM, Whittom AA, et al Cajal-body formation correlates with differential coilin phosphorylation in primary and transformed cell lines. J Cell Sci. 2009; 122: 1872–1881. 10.1242/jcs.044040 19435804 PMC2684838

[43] Prusty AB, Meduri R, Prusty BK, Vanselow J, Schlosser A, Fischer U. Impaired spliceosomal U snRNP assembly leads to Sm mRNA down-regulation and Sm protein degradation. J Cell Biol. 2017; 216(8): 2391–2407. 10.1083/jcb.201611108 28637748 PMC5551706

[44] So BR, Wan L, Zhang Z, Li P, Babiash E, Duan J, et al A U1 snRNP–specific assembly pathway reveals the SMN complex as a versatile hub for RNP exchange. Nat Struct Mol Biol. 2016; 23(3): 225–230. 10.1038/nsmb.3167 26828962 PMC4834709

[45] Braunschweig U, Barbosa-Morais NL, Pan Q, Nachman EN, Alipanahi B, Gonatopoulos-Pournatzis T, et al Widespread intron retention in mammals functionally tunes transcriptomes. Genome Res. 2014; 24(11): 1774–1786. 10.1101/gr.177790.114 25258385 PMC4216919

[46] Heins N, Cremisi F, Malatesta P, Gangemi RMR, Corte G, Price J, et al Emx2 promotes symmetric cell divisions and a multipotential fate in precursors from the cerebral cortex. Mol Cell Neurosci. 2001; 18(5): 485–502. 10.1006/mcne.2001.1046 11922140

[47] O’Leary DDM, Chou S-J, Sahara S. Area patterning of the mammalian cortex. neuron. 2007; 56(2): 252–269. 10.1016/j.neuron.2007.10.010 17964244

[48] Yoshida M, Suda Y, Matsuo I, Miyamoto N, Takeda N, Kuratani S, et al Emx1 and Emx2 functions in development of dorsal telencephalon. Development. 1997; 124(1): 101–111. 9006071 10.1242/dev.124.1.101

[49] Brunelli S, Faiella A, Capra V, Nigro V, Simeone A, Cama A, et al Germline mutations in the homeobox gene EMX2 in patients with severe schizencephaly. Nat Genet. 1996; 12(1): 94–96. 10.1038/ng0196-94 8528262

[50] Bonnal S, Vigevani L, Valcárcel J. The spliceosome as a target of novel antitumour drugs. Nat Rev Drug Discov. 2012; 11(11): 847–859. 10.1038/nrd3823 23123942

[51] Licatalosi DD, Darnell RB. Splicing regulation in neurologic disease. Neuron. 2006; 52(1): 93–101. 10.1016/j.neuron.2006.09.017 17015229

[52] Saltzman AL, Pan Q, Blencowe BJ. Regulation of alternative splicing by the core spliceosomal machinery. Genes Dev. 2011; 25(4): 373–384. 10.1101/gad.2004811 21325135 PMC3042160

[53] Jia Y, Mu John C, Ackerman Susan L. Mutation of a U2 snRNA gene causes global disruption of alternative splicing and neurodegeneration. Cell. 2012; 148(1–2): 296–308. 10.1016/j.cell.2011.11.057 22265417 PMC3488875

[54] Bezzi M, Teo SX, Muller J, Mok WC, Sahu SK, Vardy LA, et al Regulation of constitutive and alternative splicing by PRMT5 reveals a role for Mdm4 pre-mRNA in sensing defects in the spliceosomal machinery. Genes Dev. 2013; 27(17): 1903–1916. 10.1101/gad.219899.113 24013503 PMC3778243

[55] Bedford MT, Clarke SG. Protein Arginine Methylation in Mammals: Who, What, and Why. Mol Cell. 2009; 33(1): 1–13. 10.1016/j.molcel.2008.12.013 19150423 PMC3372459

[56] Friesen WJ, Massenet S, Paushkin S, Wyce A, Dreyfuss G. SMN, the product of the spinal muscular atrophy gene, binds preferentially to dimethylarginine-containing protein targets. Mol Cell. 2001; 7(5): 1111–1117. 10.1016/s1097-2765(01)00244-1 11389857

[57] Yeo G, Holste D, Kreiman G, Burge CB. Variation in alternative splicing across human tissues. Genome Biol. 2004; 5(10): R74 10.1186/gb-2004-5-10-r74 15461793 PMC545594

[58] Grosso AR, Gomes AQ, Barbosa-Morais NL, Caldeira S, Thorne NP, Grech G, et al Tissue-specific splicing factor gene expression signatures. Nucleic Acids Res. 2008; 36(15): 4823–4832. 10.1093/nar/gkn463 18653532 PMC2528195

[59] Yap K, Lim ZQ, Khandelia P, Friedman B, Makeyev EV. Coordinated regulation of neuronal mRNA steady-state levels through developmentally controlled intron retention. Genes Dev. 2012; 26(11): 1209–1223. 10.1101/gad.188037.112 22661231 PMC3371409

[60] Wong Justin JL, Ritchie W, Ebner Olivia A, Selbach M, Wong Jason WH, Huang Y, et al Orchestrated intron retention regulates normal granulocyte differentiation. Cell. 2013; 154(3): 583–595. 10.1016/j.cell.2013.06.052 23911323

[61] Ge Y, Porse BT. The functional consequences of intron retention: alternative splicing coupled to NMD as a regulator of gene expression. BioEssays. 2014; 36(3): 236–243. 10.1002/bies.201300156 24352796

[62] Peng G, Westerfield M. Lhx5 promotes forebrain development and activates transcription of secreted Wnt antagonists. Development. 2006; 133(16): 3191 10.1242/dev.02485 16854974

[63] Peukert D, Weber S, Lumsden A, Scholpp S. Lhx2 and Lhx9 determine neuronal differentiation and compartition in the caudal forebrain by regulating Wnt signaling. PLoS Biol. 2011; 9(12): e1001218 10.1371/journal.pbio.1001218 22180728 PMC3236734

[64] Suda Y, Hossain ZM, Kobayashi C, Hatano O, Yoshida M, Matsuo I, et al Emx2 directs the development of diencephalon in cooperation with Otx2. Development. 2001; 128(13): 2433–2450. 11493561 10.1242/dev.128.13.2433

[65] Weiss J, Hurley LA, Harris RM, Finlayson C, Tong M, Fisher LA, et al ENU mutagenesis in mice identifies candidate genes for hypogonadism. Mamm Genome. 2012; 23(5–6): 346–355. 10.1007/s00335-011-9388-5 22258617 PMC3358541

[66] Westerfield M. The zebrafish Book: a guide for the laboratory use of zebrafish (*Danio Rerio*). University of Oregon Press; 1995.

[67] Fröhler S, Kieslich M, Langnick C, Feldkamp M, Opgen-Rhein B, Berger F, et al Exome sequencing helped the fine diagnosis of two siblings afflicted with atypical Timothy syndrome (TS2). BMC Med Genet. 2014; 15: 1–6. 24773605 10.1186/1471-2350-15-48 PMC4038115

[68] Wang Y, Gogol-Döring A, Hu H, Fröhler S, Ma Y, Jens M, et al Integrative analysis revealed the molecular mechanism underlying RBM10-mediated splicing regulation. EMBO Mol Med. 2013; 5(9): 1431–1442. 10.1002/emmm.201302663 24000153 PMC3799496

[69] Meister G, Hannus S, Plöttner O, Baars T, Hartmann E, Fakan S, et al SMNrp is an essential pre-mRNA splicing factor required for the formation of the mature spliceosome. EMBO J. 2001; 20(9): 2304–2314. 10.1093/emboj/20.9.2304 11331595 PMC125440

[70] BOCHNIG P, REUTER R, BRINGMANN P, LÜHRMANN R. A monoclonal antibody against 2,2,7-trimethylguanosine that reacts with intact, class U, small nuclear ribonucleoproteins as well as with 7-methylguanosine-capped RNAs. Eur J Biochem. 1987; 168(2): 461–467. 10.1111/j.1432-1033.1987.tb13439.x 2959477

[71] Lerner EA, Lerner MR, Janeway CA Jr., Steitz JA. Monoclonal antibodies to nucleic acid-containing cellular constituents: probes for molecular biology and autoimmune disease. PNAS. 1981; 78(5): 2737–2741. 10.1073/pnas.78.5.2737 6789322 PMC319432

