# Supplementary material for: A missense mutation in SNRPE linked to non-syndromal microcephaly interferes with U snRNP assembly and pre-mRNA splicing
Source: PLoS Genet. 2019 Oct 31;15(10):e1008460. doi: 10.1371/journal.pgen.1008460 (PMC6850558; doi:10.1371/journal.pgen.1008460)
Supplement: S1 Text — (DOCX) [file pgen.1008460.s001.docx]

**Supporting information:**

**Clinical information of the patient**

The index patient is the first child of non-consanguineous healthy parents of German descent. Pregnancy and delivery were uneventful with mild microcephaly at birth (32 cm, -2.78 SD, < 1. centile) and normal birth weight (3185 g, -1 SD, 16. centile) and length (50 cm, -1.09 SD, 14. centile). At last follow-up at 15 years-of-age, microcephaly was prominent with a head circumference of 50 cm (-4.23 SD, < 1. centile; other anthropometric values were normal). Development delay was noted at the age of 2 years, and intellectual disability was diagnosed later with an IQ of 72 (WISC-IV, Wechsler Intelligence Scale for Children, Fourth Edition). The child attended a school for children with special needs, was able to read and write simple sentences and perform simple additions and subtractions, was able to perform simple tasks as daily life, but had fine motor function difficulties. Heterotopias but no further gross brain malformations were identified on cranial MRI. Further diagnostic work-up revealed an accessory spleen, an incomplete right bundle branch block and an IgA deficiency (Table S1). Results of genetic analyses (karyogram, array-CGH, fragile X, panel sequencing of microcephaly/epilepsy genes MCPH1, ASPM, WDR62, CDK5RAP2, CEP152, CENPJ, STIL, ATR, BUB1B, CASK, LIG4, MED17, NHEJ1, PCNT, PNKP, PQBP1, RARS2, SLC25A19, TSEN2, TSEN34, TSEN54, VRK1, ARHGEF9, ARX, ATP6AP2, ATRX, CDKL5, CNKSR2, CUL4B, DCX, FGD1, GPC3, GRIA3, HSD17B10, KDM5C, MED12, OFD1, OPHN1, PAK3, PCDH19, PHF6, PLP1, RAB39B, SLC9A6, SMC1A, SMS, SRPX2) and metabolic screening were normal.
