# Supplementary figures and images for: A missense mutation in SNRPE linked to non-syndromal microcephaly interferes with U snRNP assembly and pre-mRNA splicing
Source: PLoS Genet. 2019 Oct 31;15(10):e1008460. doi: 10.1371/journal.pgen.1008460 (PMC6850558; doi:10.1371/journal.pgen.1008460)

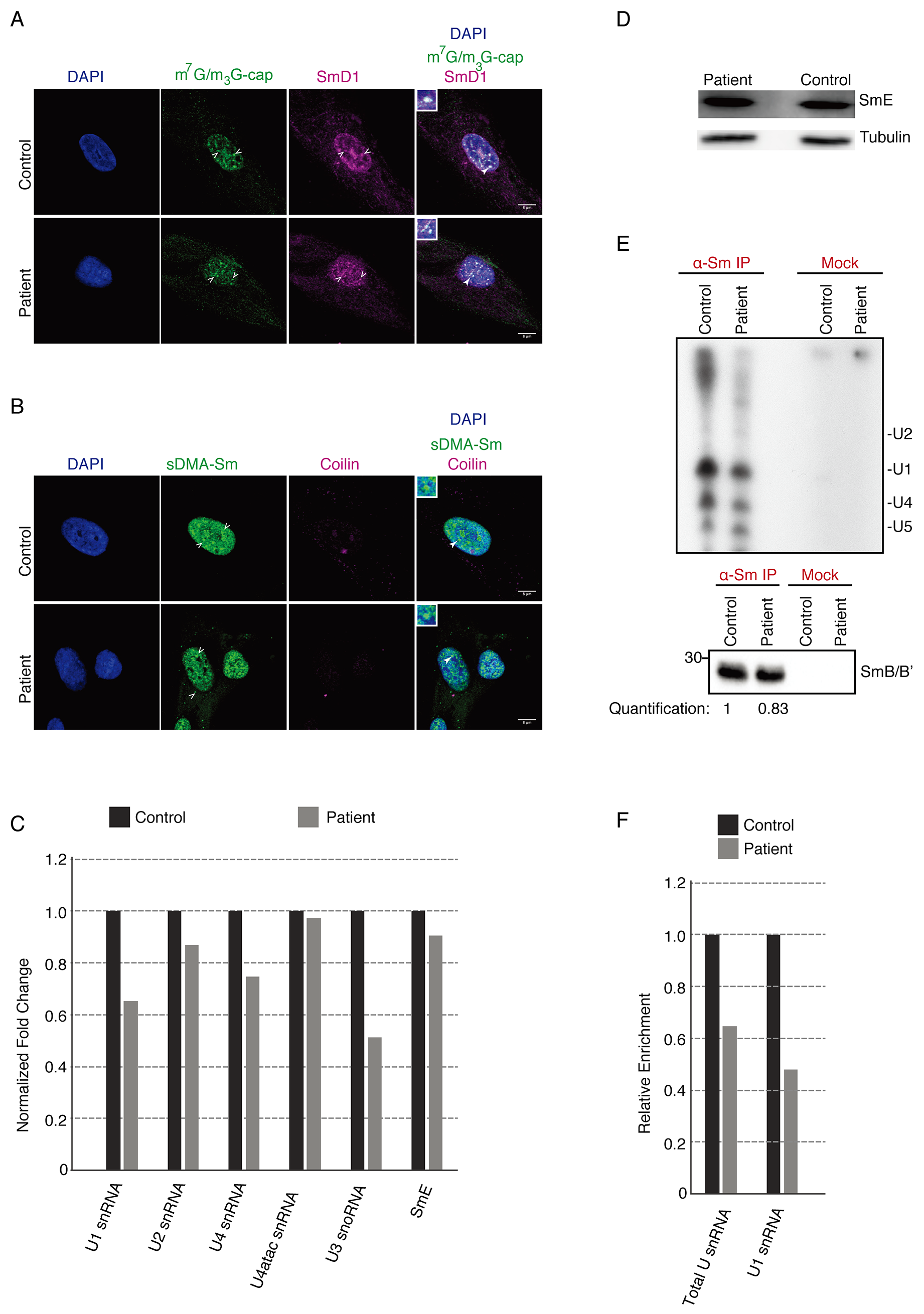

Supplement: S1 Fig — (A-B), Indirect immunofluorescence and confocal microscopy of control and patient fibroblasts. Empty white arrowheads indicate localization pattern observed and filled white arrowheads indicate zoomed in region shown in the overlay inset. (A), Co-staining with DAPI (blue), m3G/m7G cap of U snRNA (green) and SmD1 (magenta). Control fibroblasts (top panel) show abundant U snRNPs in nuclear speckles and both SmD1 and U snRNAs are predominantly absent from the cytoplasm. In patient fibroblasts (bottom panel) though there is an excellent co-localizaiton of U snRNAs and SmD1, there is a decrease in their nuclear abundance and there is an increase in their cytoplasmic localization. (B), Indirect immunofluorescence and confocal microscopy of DAPI (blue), symmetrically dimethylated (sDMA)-Sm proteins (green) and coilin (magenta). In comparison to the control fibroblasts (top panel), the patient cells (bottom panel) have reduced Sm proteins in the nucleus and an increased cytoplasmic retention. Coilin foci is not present in the images as primary cells lacks CBs. (C), Quantitative real-time PCR analysis of snRNAs and SmE in control (black bars) and patient (gray bars) fibroblasts from two independent biological replicates. (D), The SmE protein expression level in patient and control fibroblasts was checked by western blotting. The tubulin was used as loading control. (E), Immunoprecipitation of Sm proteins from control and patient fibroblasts (bottom panel, western blotting) and autoradiography (top panel) after 3’-end labeling of coprecipitated RNA. Mock indicates immunoprecipitation control without any antibody coupled to the beads. (F), Quantification of autoradiography in E; control in black and patient in gray, from two independent biological replicates. (TIF) [file pgen.1008460.s002.tif]

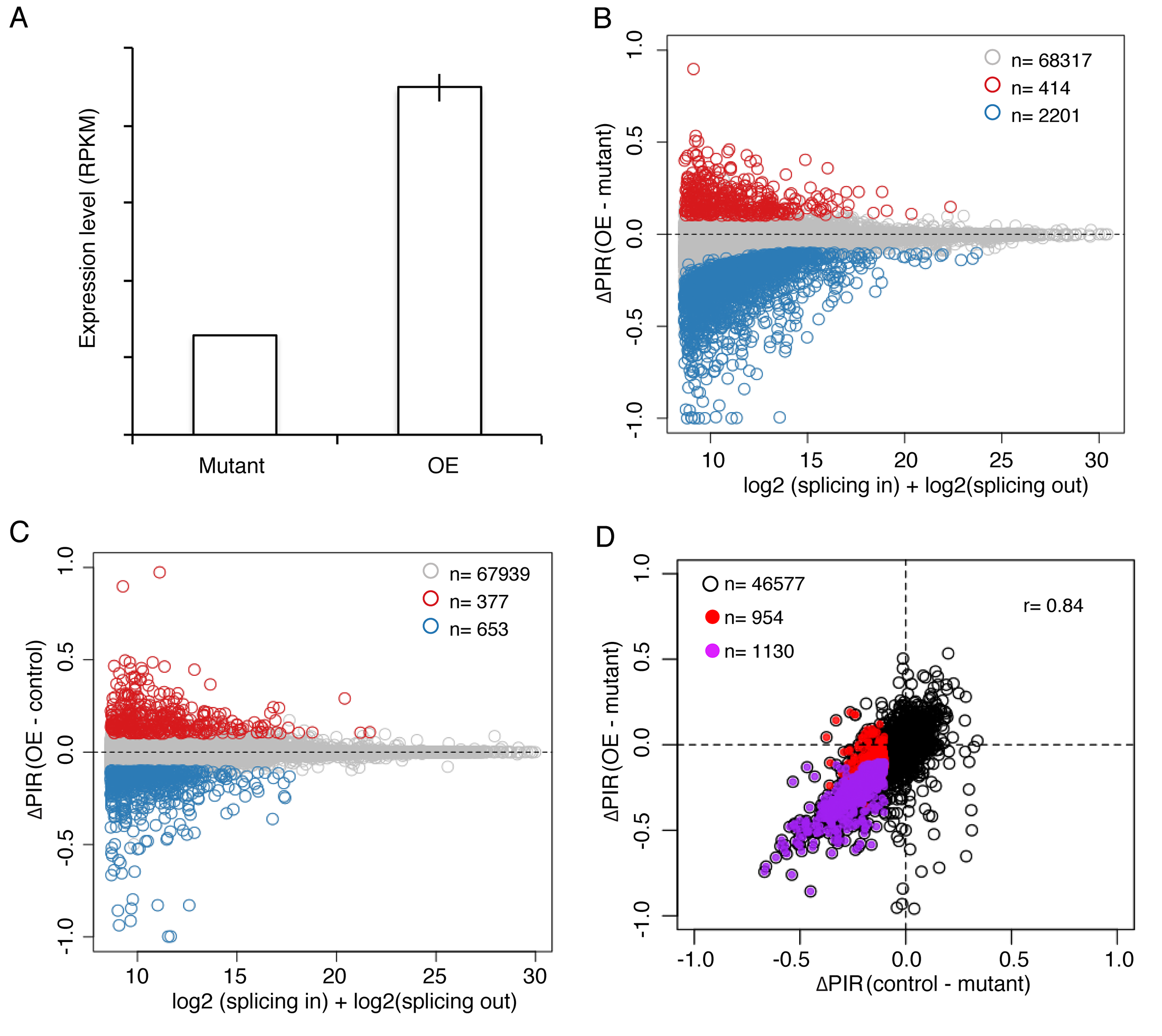

Supplement: S2 Fig — (A), Wild type SmE protein was successfully overexpressed in the patient fibroblast cells. The expression level was estimated based on RNA-seq data. (B), The MA plot compares the intron retention in the patient fibroblast cells with to those without overexpression of wild type SmE protein; X axis, log2 transformed the product of splicing in and splicing out reads number for each intron; Y axis, difference in percentage of intron retention (PIR) between the patient fibroblast cells with overexpression of wild type SmE protein (OE) and those without (mutant). (C), The MA plot compares the intron retention between the patient fibroblast cells with overexpression of wild type SmE to fibroblast cells from healthy control (control). (D), The scatter plot illustrates the PIR changes between healthy control vs mutant (X axis) and OE vs mutant (Y axis). (TIF) [file pgen.1008460.s003.tif]

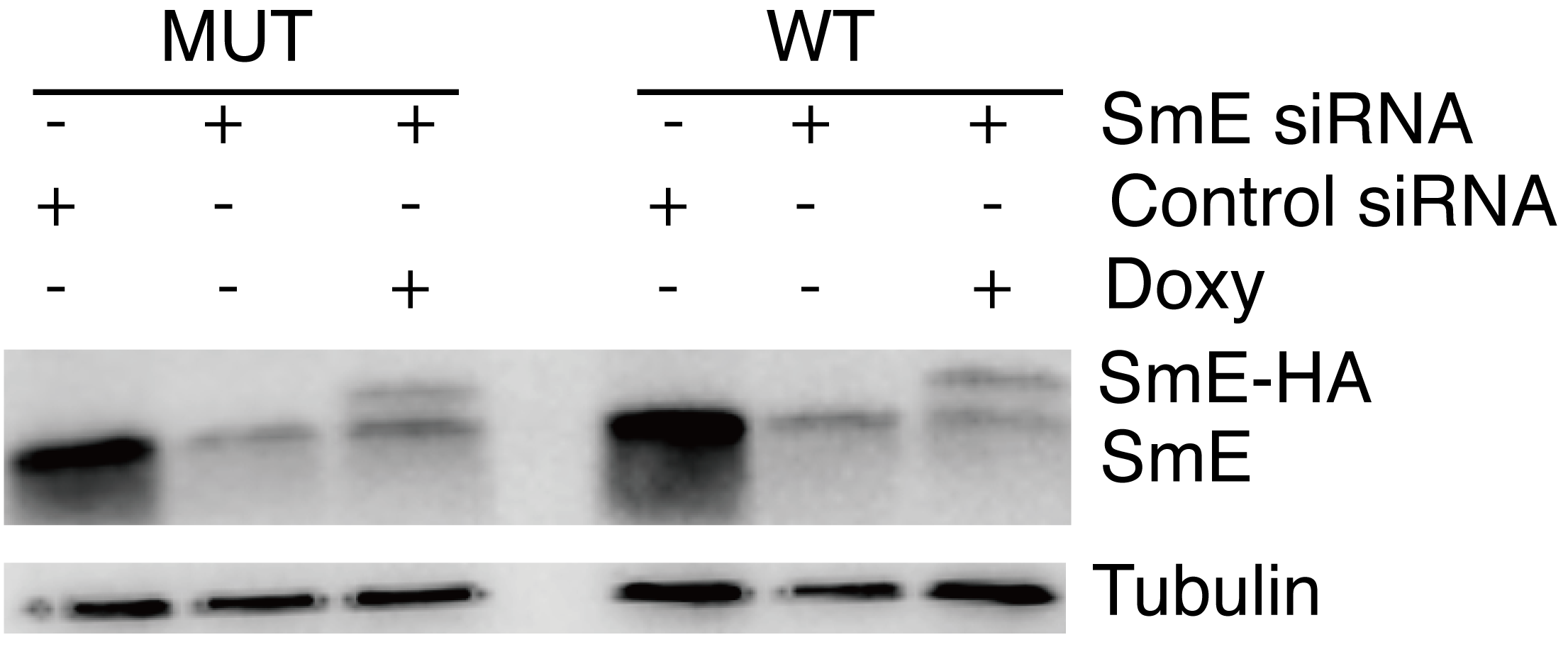

Supplement: S3 Fig — Western blot analysis shows that the endogenous SmE can be specifically depleted by SmE siRNA, targeting to the 3’ UTR region, and the exogenous HA-tagged SmE protein can be efficiently induced. The β-tubulin is used as loading control. (TIF) [file pgen.1008460.s004.tif]

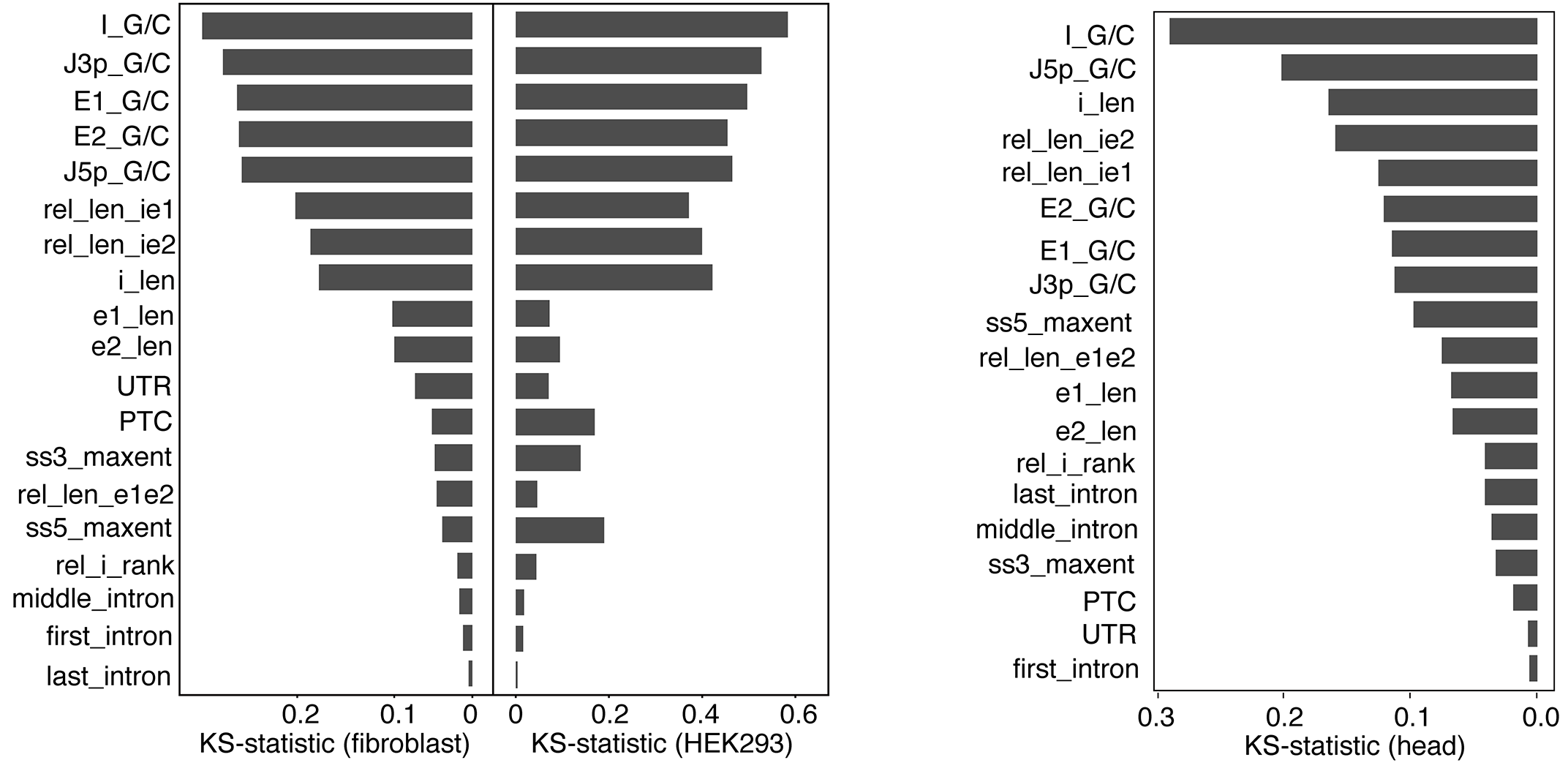

Supplement: S4 Fig — The features were compared between group 1 and group 2 (left panel); between group 3 and group 4 (middle panel); between group 5 and group 6 (right panel). The GC content is the most significantly enriched feature among all the three comparisons. Group 1: introns with increased retention in the patient fibroblast cells vs healthy control fibroblast cells (adjusted p < 0.05, delta PIR > 0.1); Group 2: introns without increased retention in the patient fibroblast cells vs healthy control fibroblast (delta PIR < 0.05, p > 0.05), this group serves as background for group 1; Group 3: introns with increased retention in HEK293 upon SmE knockdown vs control HEK293 (adjusted p < 0.05, delta PIR > 0.1); Group 4: introns without increased retention in HEK293 upon SmE knockdown vs control HEK293 (delta PIR < 0.05, p > 0.05), this group serves as background for group 3; Group 5: introns with increased retention in zebrafish upon SmE knockdown vs control (adjusted p < 0.05, delta PIR > 0.1); Group 6: introns without increased retention in zebrafish upon SmE knockdown vs control (delta PIR < 0.05, p > 0.05), this group serves as background for group 5. (TIF) [file pgen.1008460.s005.tif]

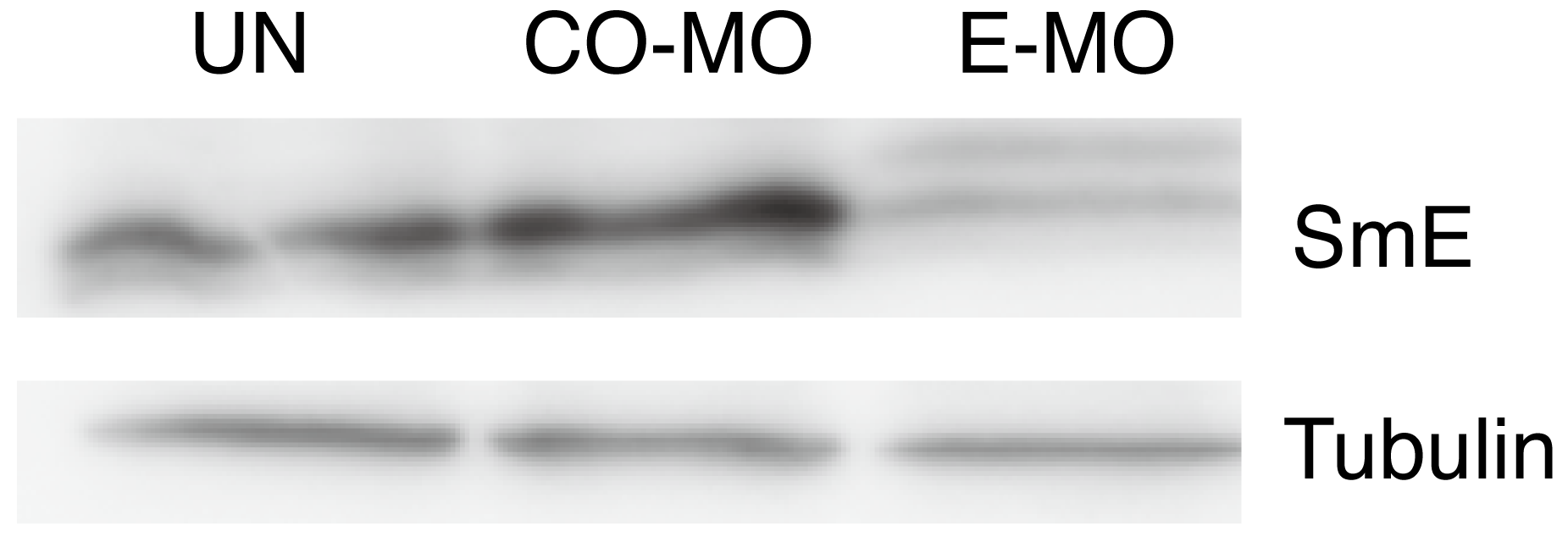

Supplement: S5 Fig — Western blot analysis shows that the endogenous zSmE can be specifically depleted by SmE morpholino, targeting to the translation initiation site. The β-tubulin is used as loading control. UN, un-injection; CO-MO, control morpholino; E-MO, SmE morpholino. (TIF) [file pgen.1008460.s006.tif]
